# Activation of podocyte Notch mediates early *Wt1* glomerulopathy

**DOI:** 10.1016/j.kint.2017.11.014

**Published:** 2018-04

**Authors:** Rowan I. Asfahani, Mona M. Tahoun, Eve V. Miller-Hodges, Jack Bellerby, Alex K. Virasami, Robert D. Sampson, Dale Moulding, Neil J. Sebire, Peter Hohenstein, Peter J. Scambler, Aoife M. Waters

**Affiliations:** 1 Programme of Developmental Biology of Birth Defects, Great Ormond Street Institute of Child Health, University College of London, London, UK; 2 Clinical and Chemical Pathology Department, Faculty of Medicine, Alexandria University, Alexandria, Egypt; 3 MRC Institute of Genetics and Molecular Medicine, University of Edinburgh, Edinburgh, Scotland; 4 Great Ormond Street Hospital NHS Foundation Trust, London, UK; 5 Institute of Ophthalmology, University College of London, London, UK; 6 The Roslin Institute, Edinburgh, Scotland

**Keywords:** albuminuria, focal segmental glomerulosclerosis, nephrotic syndrome, podocyte

## Abstract

The Wilms' tumor suppressor gene, *WT1*, encodes a zinc finger protein that regulates podocyte development and is highly expressed in mature podocytes. Mutations in the *WT1* gene are associated with the development of renal failure due to the formation of scar tissue within glomeruli, the mechanisms of which are poorly understood. Here, we used a tamoxifen-based CRE-LoxP system to induce deletion of *Wt1* in adult mice to investigate the mechanisms underlying evolution of glomerulosclerosis. Podocyte apoptosis was evident as early as the fourth day post-induction and increased during disease progression, supporting a role for *Wt1* in mature podocyte survival. Podocyte Notch activation was evident at disease onset with upregulation of *Notch1* and its transcriptional targets, including *Nrarp*. There was repression of podocyte *FoxC2* and upregulation of *Hey2* supporting a role for a Wt1/FoxC2/Notch transcriptional network in mature podocyte injury. The expression of cleaved Notch1 and HES1 proteins in podocytes of mutant mice was confirmed in early disease. Furthermore, induction of podocyte HES1 expression was associated with upregulation of genes implicated in epithelial mesenchymal transition, thereby suggesting that HES1 mediates podocyte EMT. Lastly, early pharmacological inhibition of Notch signaling ameliorated glomerular scarring and albuminuria. Thus, loss of *Wt1* in mature podocytes modulates podocyte Notch activation, which could mediate early events in *WT1*-related glomerulosclerosis.

Glomerulosclerosis accounts for 5% to 10% of pediatric and adult end-stage kidney disease and recurs in 15% to 30% of patients following kidney transplantation.^1^ Mutations in genes encoding transcription factors which regulate podocyte differentiation have been implicated in human glomerulosclerosis and include Wilms’ tumor 1 (WT1).^2^, ^3^, ^4^, ^5^, ^6^, ^7^ WT1 encodes a nuclear protein containing 4 zinc fingers that bind DNA and RNA and is highly expressed in mature podocytes.^8^, ^9^, ^10^, ^11^ Mutations in the regions involved in DNA binding or zinc finger formation have been reported in patients with Denys-Drash syndrome, a disorder associated with infantile diffuse mesangial sclerosis, gonadal dysgenesis, and Wilms’ tumor.^12^, ^13^, ^14^ Mutations disrupting an alternative splice donor site in intron 9, results in Frasier syndrome, a disorder associated with focal segmental glomerulosclerosis (FSGS), predisposition to gonadoblastoma, and male pseudohermaphroditism.^15^, ^16^ Furthermore, mutations in *WT1* have been reported in isolated primary steroid-resistant nephrotic syndrome, supporting a role for aberrant Wt1 function in the pathogenesis of glomerulosclerosis.^5^, ^7^

Mechanisms underlying the development of *WT1*-related glomerulosclerosis are poorly understood with no effective treatments available. Recent studies employing chromatin immunoprecipitation sequencing, RNA sequencing, exon array, and bioinformatic analyses have shown a specific role for WT1 in regulating the podocyte-specific transcriptome. WT1 can bind to both promoters and enhancers of 18 known podocytopathy genes.^17^, ^18^ Mutations in *WT1* could lead to altered transcriptional regulation of these genes, which is necessary for podocyte differentiation and function, and contribute to the pathogenesis of glomerulosclerosis.^17^, ^18^

Genes regulating podocyte differentiation have also been implicated in the pathogenesis of *WT1*-related disease. *De novo* expression of PAX2 protein and mRNA, a paired box transcription factor repressed by WT1 during nephrogenesis, has been observed in podocytes of Denys-Drash syndrome patient biopsies.^19^ Furthermore, re-expression of Pax2 in podocytes, cell cycle re-entry, and reduced expression of podocyte proteins such as nephrin and α-actinin-4 have been reported in heterozygous *Wt1*^*tmT396/+*^ mice with glomerulosclerosis by 8 months of age.^20^ These findings suggested that *Wt1*^*tmT396/+*^ podocytes dedifferentiate and revert to an immature phenotype during disease progression.^20^

During glomerulogenesis, podocyte fate induction is regulated by Notch, a highly conserved and ubiquitous pathway that transduces short-range signals between neighboring cells.^21^, ^22^, ^23^, ^24^ Ligand binding initiates regulated intramembrane proteolysis with subsequent nuclear translocation of the Notch intracellular domain (NICD) where it associates with RBPJ-κ, a DNA-binding protein, and promotes transcription of target genes (e.g., hairy enhancer of split [Hes]), which regulates tissue-specific differentiation. *Notch1*, *Notch2,* and downstream transcriptional targets *Hes1* and *Hey1* are expressed in podocyte precursors in the S-shaped body and are down-regulated during terminal differentiation.^25^, ^26^ Ectopic podocyte Notch activation in differentiating and mature podocytes is associated with both diffuse mesangial sclerosis and FSGS, respectively, the latter being mediated by p53-induced podocyte apoptosis and the former associated with *de novo* Pax2 expression.^27^, ^28^ Given the interplay between WT1 and Notch during glomerulogenesis and the fact that both diffuse mesangial sclerosis and FSGS phenotypes occur with mutations in *WT1* as well as ectopic podocyte Notch activation, we hypothesized a role for podocyte Notch activation in the development of glomerulosclerosis related to loss of Wt1 function.

In a model where *Wt1* was deleted in mature podocytes, using a tamoxifen-based CRE-LoxP system, we observed increased podocyte loss during the development of glomerulosclerosis. At disease onset, we found upregulation of Notch pathway transcripts in mutant podocytes. At the same time point, we observed repression of *FoxC2* and upregulation of *Hey2* transcripts in primary mutant podocytes. Cleaved Notch1 and HES1 proteins were evident in mutant podocytes at disease manifestation. Induction of podocyte HES1 expression *in vitro* was associated with increased expression of *Slug* and *Snail* transcripts, genes implicated in epithelial to mesenchyme transition. Pharmacological inhibition of Notch with gamma-secretase inhibitors at disease onset rescued the severity of glomerulosclerosis and albuminuria. These data support a role for early Notch activation in the manifestation of *Wt1* glomerulopathy, which may be mediated via repression of podocyte *FoxC2*.

## Results

### Early glomerulosclerosis is evident at 5 days post tamoxifen induction in *CAGG-CreER*^*TM+/−*^;*Wt1*^*f/f*^ transgenic mice

**Figure 1:**
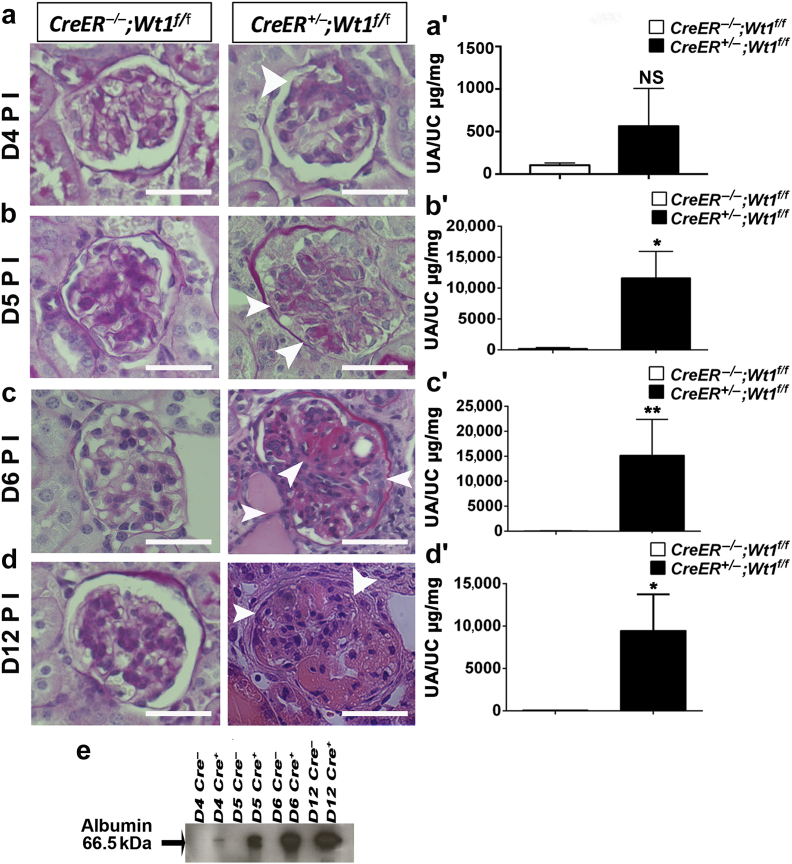
**Temporal induction of glomerulosclerosis in *CAGG-CreER***^***TM−/+***^***;Wt1***^***f/f***^**transgenic mice following tamoxifen induction (a–d).** (**a**) At day (D) 4 postinduction (PI), glomeruli of *CAGG-CreER*^*TM−/+*^*;Wt1*^*f/f*^ transgenic mice (mutants) are morphologically similar to their controls, *CAGG-CreER*^*TM−/−*^*;Wt1*^*f/f*^ transgenic mice. (See Supplementary Figure S2 for analysis of heterozygous *CAGG-CreER*^*TM−/+*^*;Wt1*^*f/+*^ littermates and Supplementary Figure S3 for analysis of severity of glomerulosclerosis). Bars = 50 μm. (**a′**) Quantitative graph showing mean urine albumin-creatinine ratio (μg/mg) ±SEM at D4 PI controls versus mutants (*n* = 14 vs. *n* = 10); 104.4 ± 25.71 versus 563.5 ± 443, *P* = 0.21, Student *t*-test. (**b**) At D5 PI, mutant mice exhibit segmental glomerulosclerosis compared with control mice (for quantitative analysis, see also Supplementary Figure S3). (**b′**) Quantitative graph showing mean urine albumin-creatinine ratio (μg/mg) at D5 PI in controls versus mutants (*n* = 14 vs. *n* = 14); 217 ± 157.5 versus 11,654 ± 4304, *P = 0.01, Student *t*-test. (**c**) At D6 PI, more extensive glomerulosclerosis is evident in mutants compared with control mice with hyaline-filled tubules. (**c′**) Quantitative graph showing mean urine albumin-creatinine ratio (μg/mg) at D6 PI in controls versus mutants (*n* = 5 vs. *n* = 6); 79.8 ± 29.8 versus 15,202 ± 7210, **P = 0.004, Student *t*-test. (**d**) By D12 PI, glomeruli of mutant mice exhibit global glomerulosclerosis, hyaline-filled tubules, and pyknotic podocyte nuclei with extensive tubulointerstitial disease (see also Supplementary Figure S5). (**d′**) Quantitative graph showing mean urine albumin-creatinine ratio (μg/mg) at D12 PI controls versus mutants (*n* = 7 vs. *n* = 7); 76.8 ± 13.9 versus 9469 ± 4279, *P = 0.04, Student *t*-test. (**e**) Western blot analysis from D4, D5, D6, and D12 PI shows evidence of increased albuminuria in mutant urine during disease progression in *CAGG-CreER*^*TM−/+*^*;Wt1*^*f/f*^ transgenic mice compared with Cre-negative control mice. To optimize viewing of this image, please see the online version of this article at www.kidney-international.org.

**Figure S1:**
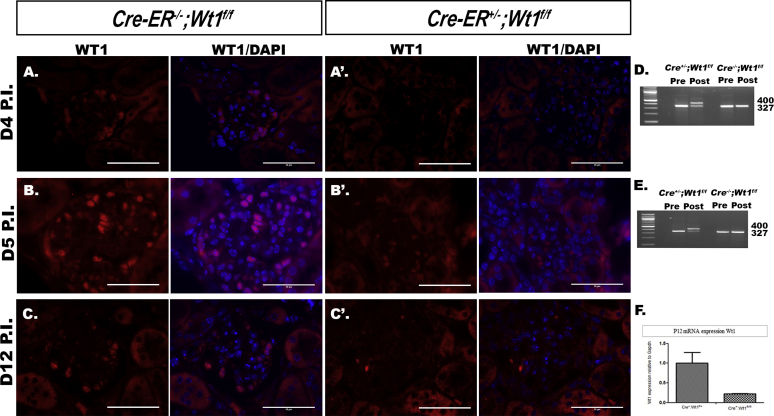
Wilms’ tumor 1 (*Wt1)* is deleted at day (D) 4, D5, and D12 postinduction (PI) of tamoxifen. (**A–C′**) Representative micrographs of WT1 protein expression in glomeruli following immunofluorescence labeling of *Cre*^*TM−/−*^*;Wt1*^*f/f*^ control mouse kidney tissue sections versus *Cre*^*TM+/−*^*;Wt1*^*f/f*^ mutants. Bars = 50 μm. Images were counterstained with 4′,6-diamidino-2-phenylindole (DAPI). (**A,A′**) D4 PI controls show WT1 protein expression as indicated by red arrows (**A**) in comparison to mutants, where WT1 protein expression is reduced (**A′**). (**D**) DNA expression of *Wt1* PI of tamoxifen following polymerase chain reaction analysis. Recombination is present in the mutants, but no recombination is seen in controls. (**B,B′**) D5 PI micrographs highlighting WT1 protein expression in controls, as indicated by arrows (**B**), versus mutants, where no WT1 protein is observed (**B′**). (**E**) Polymerase chain reaction analysis of *Wt1* genotyping PI of tamoxifen in mutants versus controls. *Wt1* amplicon is reduced and recombination is shown in the mutant. (**C,C′**) Representative micrographs of glomeruli following immunofluorescent labeling of podocytes with WT1 in D12 PI sections. WT1 protein is present in controls, as indicated by the red arrows (**C**), versus mutants, where WT1 is absent (**C′**). (**F**) Quantitative real-time polymerase chain reaction graph highlighting mRNA expression of *Wt1* relative to *Gapdh* showing a reduction of *Wt1* in the mutant versus control.

*Wt1* deletion in mature podocytes results in glomerulosclerosis with compromised renal function by day 7 post tamoxifen induction in adult *CAGG-CreER*^*TM+/−*^;*Wt1*^*f/f*^ transgenic mice.^29^ To investigate events leading to the induction of disease in these mice, we first determined the earliest point at which we could detect glomerulosclerosis after *Wt1* deletion. Tamoxifen was administered for 3 consecutive days by i.p. injection to 5-week-old *CAGG-CreER*^*TM+/−*^;*Wt1*^*f/f*^ transgenic mice and mice were nephrectomized at 4, 5, 6, and 12 days following injection. Successful *Wt1* deletion was demonstrated by recombination polymerase chain reaction (PCR) and the reduction of Wt1 expression in glomeruli (Supplementary Figure S1). Following light microscopy analysis of periodic acid–Schiff (PAS)-stained kidney sections, we determined the severity of glomerulosclerosis by a semiquantitative analysis at each time point in *CAGG-CreER*^*TM+/−*^;*Wt1*^*f/f*^ mutants, *CAGG-CreER*^*TM−/−*^*;Wt1*^*f/f*^ controls, and heterozygous *CAGG-CreER*^*TM+/−*^*;Wt1*^*f/+*^ mice (Figure 1a–d).

**Figure S2:**
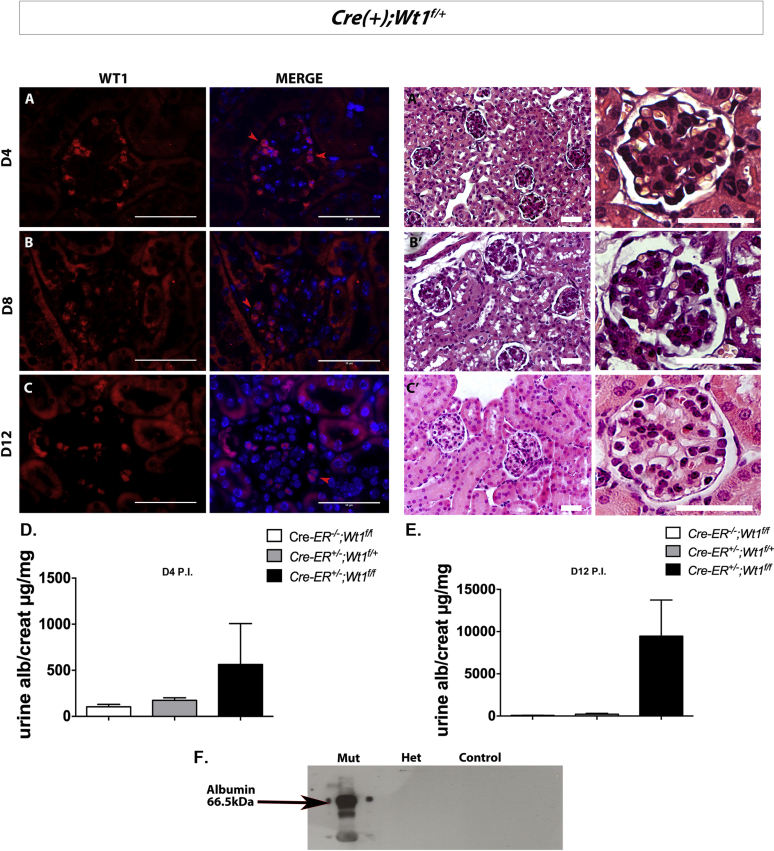
Wilms’ tumor 1 (WT1) is expressed in *Cre(+);Wt1*^*f/+*^ transgenic mice postinduction (PI) of tamoxifen. (**A-C**) Representative micrographs of glomeruli following immunofluorescent labeling of podocytes with anti-WT1 antibody in day (D) 4, D8, and D12 *Cre* ^*TM+/−*^*;WT1*^*f/+*^ heterozygotes PI. Bars = 50 μm. (**A**) At D4 PI, WT1 protein is expressed in *Cre*^*TM+/−*^*;Wt1*^*f/+*^ glomeruli following tamoxifen induction, as indicated by arrows. (**B**) WT1 protein expression is present at D8 PI in the heterozygotes, as seen by the arrows. (**C**) By D12 PI, WT1 protein is still present in the heterozygotes. (**A′,B′**) Hematoxylin and eosin–stained sections of glomeruli at D4, D8, and D12 PI. Bar = 25 μm. (**A′**) Mice exhibit healthy glomeruli with no histological abnormalities at D4 PI. (**B′**) At D8 PI, no hyaline presence or glomerulosclerosis and glomeruli are intact. (**C′**) No histological abnormalities in the glomeruli by D12 PI with no hyaline material in either the glomeruli or tubules. (**D**) Quantitative graph showing mean urine albumin-creatinine ratio (μg/mg) at D4 PI controls versus heterozygotes versus mutants (*n* = 14 vs*. n* = 8 vs*. n* = 10): 104.4 ± 25.7 versus 174.9 ± 27.0 versus 563.5 ± 443.9, *P =* 0.21, Student *t*-test. (**E**) Quantitative graph showing mean urine albumin-creatinine ratio (μg/mg) at D12 PI controls versus heterozygotes versus mutants (*n* = 8 versus *n* = 3 versus *n* = 7): 76.8 ± 13.9 versus 223.5 ± 93.8 versus 9469 ± 4279, *P* = 0.21, Student *t*-test. (**F**) Western blot analysis of urine samples of *Cre*^*TM−/−*^*;Wt1*^*f/f*^, *Cre*^*TM+/−*^*;Wt1*^*f/+*^*,* and *Cre*^*TM+/−*^*;Wt1*^*f/f*^ at D8 PI probed with anti-albumin. Albuminuria is detected in the mutant and absent in both controls (molecular weight of albumin: 66.5 kDa).

**Figure S3:**
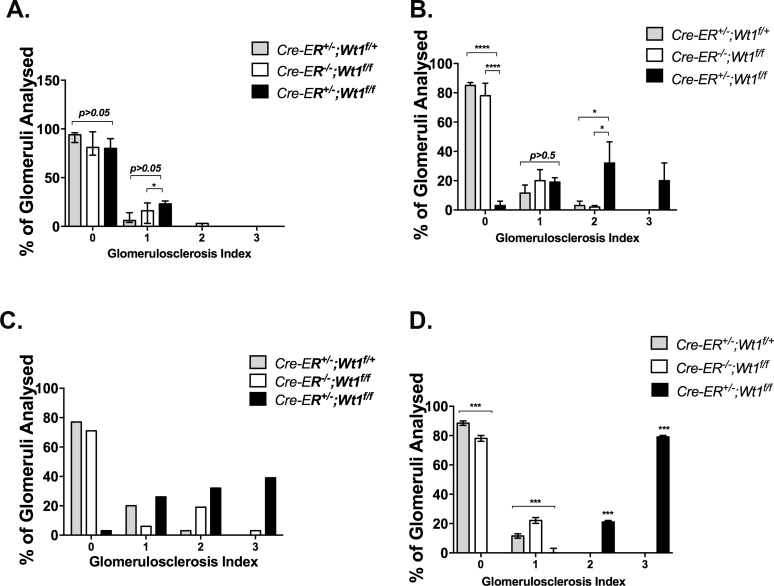
Induction and severity of glomerulosclerosis at day (D) 4, D5, D6, and D12 postinduction (PI). (**A**-**D**) Quantitative graphs of proportion of glomeruli per genotype revealing extent of glomerulosclerosis (score 0: <25% glomerulus sclerosed; score 1: 25%–50% sclerosis; score 2: 50%–75% sclerosis; score 3: >75% sclerosis). (**A**) At D4 PI, none of the transgenic mice exhibited global glomerulosclerosis (>75% sclerosis, score 3). A significant proportion of mutants exhibited segmental glomerulosclerosis (<50%, score 1) compared with controls and heterozygotes: D4 PI: *CreER*^*+/−*^*;Wt1*^*f/+*^ (heterozygotes) (*n* = 90 glomeruli, *n* = 3 mice) versus *CreER*^*−/−*^*;Wt1*^*f/f*^ (controls) versus *CreER*^*+/−*^*;Wt1*^*f/f*^ (*n* = 90 glomeruli, *n* = 3 mice) (mutants) transgenic mice. Score 0: 94% (interquartile range [IQR]: 86, 96) versus 81% (IQR: 73, 97) versus 80% (IQR: 77, 90), *P* = 0.06; score 1: 6% (IQR: 4, 14) versus 16% (IQR: 3, 24) versus 23% (IQR: 20, 26). Bars represent the median percentage of each score per genotype. Error bars represent the IQR, *P < 0.05, Mann-Whitney U test. (**B**). At D5 PI, a significant proportion of mutant glomeruli with more extensive segmental sclerosis were observed compared with controls and heterozygotes: D5 PI: *CreER*^*+/−*^*;Wt1*^*f/+*^ (*n* = 60 glomeruli) versus *CreER*^*−/−*^*;Wt1*^*f/f*^ (*n* = 90 glomeruli) versus *CreER*^*+/−*^*;Wt1*^*f/f*^ (*n* = 90 glomeruli): score 0: 85 ± 2% versus 78 ±8.5% versus 3 ± 3%,****P < 0.0001, Student *t*-test; score 1: 11.5 ± 5.5% versus 20 ± 7.6% versus 19 ± 3.1%; *P* = 0.5 (not significant); score 2: 3 ± 3% versus 2±1% versus 32 ± 14.5%, *P < 0.05; score 3: 0% versus 0% versus 20 ± 12.2%, *P < 0.05, Student *t*-test. Bars represent the mean percentage of each score per genotype. Error bars represent the SEM. ****P < 0.0001, *P < 0.05, Student *t*-test. (**C**) At D6 PI, more extensive glomerulosclerosis (40% with score 3 vs. 1% heterozygotes and 0% for controls) was evident in mutants compared with controls. (**D**) By D12 PI, the majority of glomeruli exhibited >75% glomerulosclerosis: D12 PI *CreER*^*+/−*^*;Wt1*^*f/+*^ versus *CreER*^*−/−*^*;Wt1*^*f/f*^ (*n* = 60 glomeruli) versus *CreER*^*+/−*^*;Wt1*^*f/f*^ (*n* = 61 glomeruli) transgenic mice. Score 0: 88.5% (IQR: 87, 90) versus 78% (IQR: 76, 80) versus 0% (IQR: 0, 0), ***P < 0.001, 2-way analysis of variance (ANOVA); score 1: 11.5% (IQR: 10, 13) versus 22% (IQR:0, 24) versus 0% (IQR: 0, 3), ***P < 0.001, 2-way ANOVA; score 2: 0% versus 0% versus 21% (IQR: 20, 22), ***P < 0.001, 2-way ANOVA; score 3: 0% versus 0% versus 79% (IQR: 78, 80), ***P < 0.001, 2-way ANOVA.

**Figure S4:**
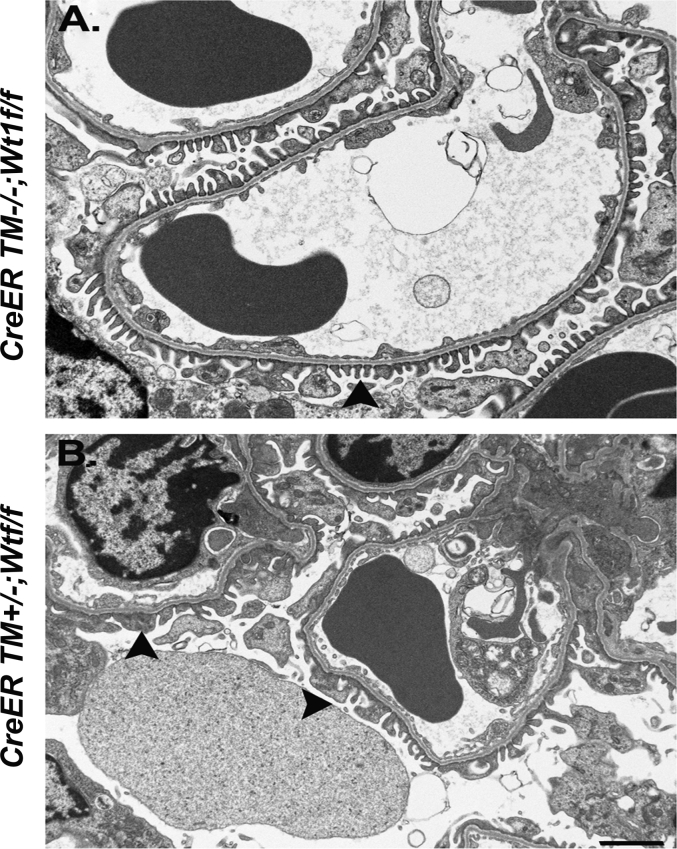
Podocyte foot process effacement at day (D) 4 postinduction (PI) in CAGG-CreERTM+/-;Wt1f/f transgenic mice. (**A,B**) Representative electron micrographs demonstrating focal foot process effacement in mutant mice (**B**) as early as D4 PI compared with control mice (**A**).

**Figure S5:**
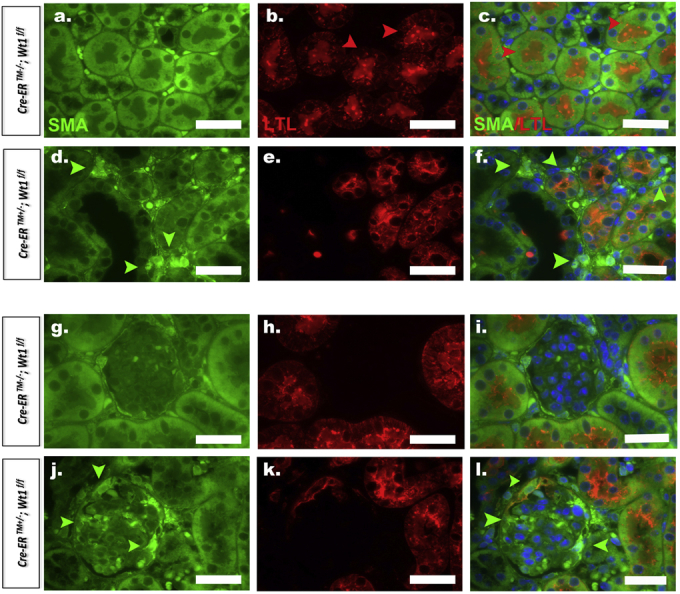
Induction of vascular smooth muscle actin expression in *CreER*^*TM+/−*^*;Wt1*^*f/f*^ transgenic mice. (**A-F**) Shown are micrographs of glomeruli following double immunofluorescence labeling of transgenic mouse kidney tissue sections with anti-α smooth muscle actin (SMA) (Alexa Fluor 488–conjugated secondary antibody) and *Lotus tetragonolobus* lectin (LTL) (Alexa Fluor 594–conjugated secondary antibody) at day (D) 12 PI. Images were counterstained with 4′,6-diamidino-2-phenylindole. Bars = 50 μm. (**A–F**) Expansion of α-SMA expression is noted in tubulointerstitial compartment in mutants (**D**) compared with controls (**A**). (**C,F**) High-power views of tubulointerstitium. α-SMA expression (green arrows) is induced in peritubular cells (red arrows, tubules) in *CreER*^*TM+/−*^*;Wt1*^*f/f*^ transgenic mice (**F**) that is not seen in control *CreER*^*TM−/−*^*;Wt1*^*f/f*^ transgenic mice (**C**). Bars = 50 μm. (**G–L**) High-power views of proximal nephron and glomeruli. Vascular SMA expression (green arrows) is observed in *CreER*^*TM+/−*^*;Wt1*^*f/f*^ transgenic mice (**L**) in clusters of glomerular cells within Bowman’s capsule, intraglomerular tuft region, and the parietal epithelium in *CreER*^*TM+/−*^*;Wt1*^*f/f*^ and not in control *CreER*^*TM−/−*^*;Wt1*^*f/f*^ transgenic mice (**G**). Bars = 50 μm.

Heterozygous mice did not develop glomerulosclerosis by day (D) 12 postinduction (PI) (Supplementary Figure S2). At D4 PI, mutants exhibited early segmental glomerulosclerosis with focal foot process effacement and a trend toward higher levels of albuminuria (*P =* 0.21) (Figure 1a and a′; Supplementary Figures S3A and S4). By D5 PI, glomerular scarring was more extensive (≥ score 2) in mutants compared with control and heterozygous mice (*P < 0.05) (Figure 1b, Supplementary Figure S3B). Progression of disease was further supported by an increase in urine albumin-creatinine ratio in mutants compared with controls (*P = 0.01) (Figure 1b′). By D6 PI, extensive glomerulosclerosis with tubules containing protein casts were observed in mutants relative to controls with increased albuminuria (Figure 1c and c′, Supplementary Figure S3C). Late stage disease with global glomerulosclerosis was observed at D12 PI (Figure 1d and e, Supplementary Figure S3D), with peritubular cells expressing vascular smooth muscle actin, consistent with progression of tubulointerstitial disease (Supplementary Figure S5). We conclude that podocyte function appears compromised within 6 days of *Wt1* deletion in mature podocytes. Therefore, investigations into the mechanisms underlying manifestation of disease should be undertaken within this time frame.

### Cleaved-Caspase-3 protein expression increases in glomeruli and podocytes of mutant mice at D5, D6, and D12 PI

**Figure 2:**
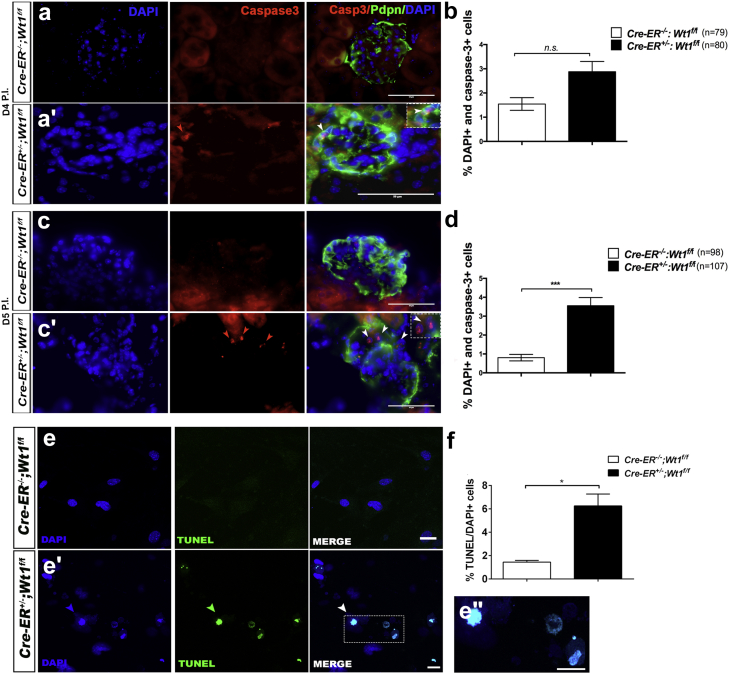
**Podocyte apoptosis in *CAGG-CreER***^***TM−/+***^***;Wt1***^***f/f***^**transgenic mice following tamoxifen administration (a,a′).** Shown are split-channel and merged micrographs of glomeruli following double immunofluorescence labeling of transgenic mouse kidney tissue sections with anti-Cleaved-Caspase-3 (Casp-3) (Alexa Fluor 594–conjugated secondary antibody) and anti-Podoplanin (Alexa Fluor 488–conjugated secondary antibody) at day (D) 4 postinduction (PI). Images were counterstained with 4′,6-diamidino-2-phenylindole (DAPI). At D4 PI, Cleaved-Caspase-3–positive, Podoplanin-positive cells were observed in DAPI-positive cells in mutant glomeruli (**a′**) and not in control glomeruli (**a′**). Bars = 25 μm. (**b**) Quantitative graphs showing mean percentage of DAPI-positive, Cleaved-Caspase-3–positive cells per glomerulus at D4 PI in control and mutant mice. Bars represent mean and error bars indicate the SEM. A nonsignificant increase was observed in the number of DAPI-positive, Cleaved-Caspase-3–positive glomerular cells in control versus mutant mice at D4 PI: controls versus mutants (*n* = 79 vs. *n* = 80 glomeruli from *n* = 3 per genotype); 1.6 ± 0.3% versus 3.0 ± 0.4%, *P* = 0.06, not significant (NS). Student *t*-test. (**c,c′**) At D5 PI, Cleaved-Caspase-3–positive (red arrows), DAPI-positive cells in mutant glomeruli stained with Podoplanin (inset, white arrows) and not in control glomeruli. (**d**) Quantitative graphs showing mean percentage of DAPI-positive, Cleaved-Caspase-3–positive, Podoplanin-positive cells per glomerulus at D5 PI in control and mutant mice: controls versus mutants (*n* = 98 vs. *n* = 107 glomeruli from *n* = 3 per genotype); 0.8 ± 0.2% versus 3.6 ± 0.4%, ***P < 0.0001, Student *t*-test. (**e,e′**) Representative micrographs following immunofluorescent labeling of terminal deoxynucleotidyltransferase–mediated 2′-deoxyuridine 5′-triphosphate nick end-labeling (TUNEL)–positive primary podocytes (Alexa Fluor 488–conjugated secondary antibody, green arrows) isolated at D6 PI from control and mutant mice. Sections were counterstained with DAPI. (**e′′**) Higher-power magnification showing a cell nucleus–expressing TUNEL-positive signal. (**f**) Quantitative graph of apoptotic cells calculating proportion of TUNEL-DAPI–positive podocytes at D6 posttamoxifen *in vitro*. Bars represent the mean, and error bars represent the SEM. *CAGG-CreER*^*−/−*^*;Wt1*^*f/f*^ (*n* = 2 samples, 10 fields quantified per sample) versus *CAGG-CreER*^*+/−*^*;Wt1*^*f/f*^ (*n* = 2 mice per genotype, 10 fields quantified per primary podocyte line per mouse); 1.4 ± 0.2% versus 6.3 ± 1.0%, *P = 0.04, Student *t*-test. See also Supplementary Figure S6 for primary podocyte caspase-3/7 staining; Supplementary Figure S7 for TUNEL staining *in vivo* at D5 PI, and Supplementary Figure S8 for Annexin V PI staining of primary podocytes for control and mutant transgenic mice. To optimize viewing of this image, please see the online version of this article at www.kidney-international.org.

**Figure S6:**
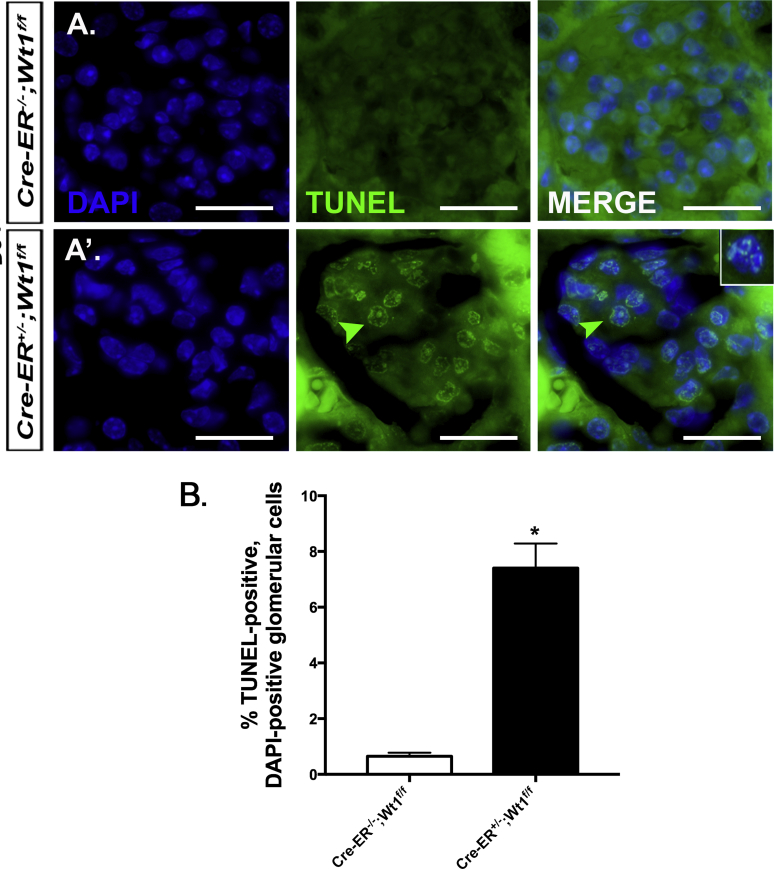
Terminal deoxynucleotidyltransferase–mediated 2′-deoxyuridine 5′-triphosphate nick end-labeling (TUNEL)-positive cells in glomeruli of *CAGG-CreER*^*TM+/−*^*;Wt1*^*f/f*^ transgenic mice. (**A,A′**) TUNEL-positive and 4′,6-diamidino-2-phenylindole (DAPI)-positive cells (inset) in mutant mice (**A′**) at day 5 postinduction compared with controls (**A**). (**B**) Quantitative graph showing increased proportion of median number of TUNEL-positive podocytes. Bars represent the median of each group. Error bars represent interquartile ranges, *P < 0.0001, Mann-Whitney U test. See also Figure 2c for TUNEL-positive primary podocytes of mutant versus control mice.

**Figure S7:**
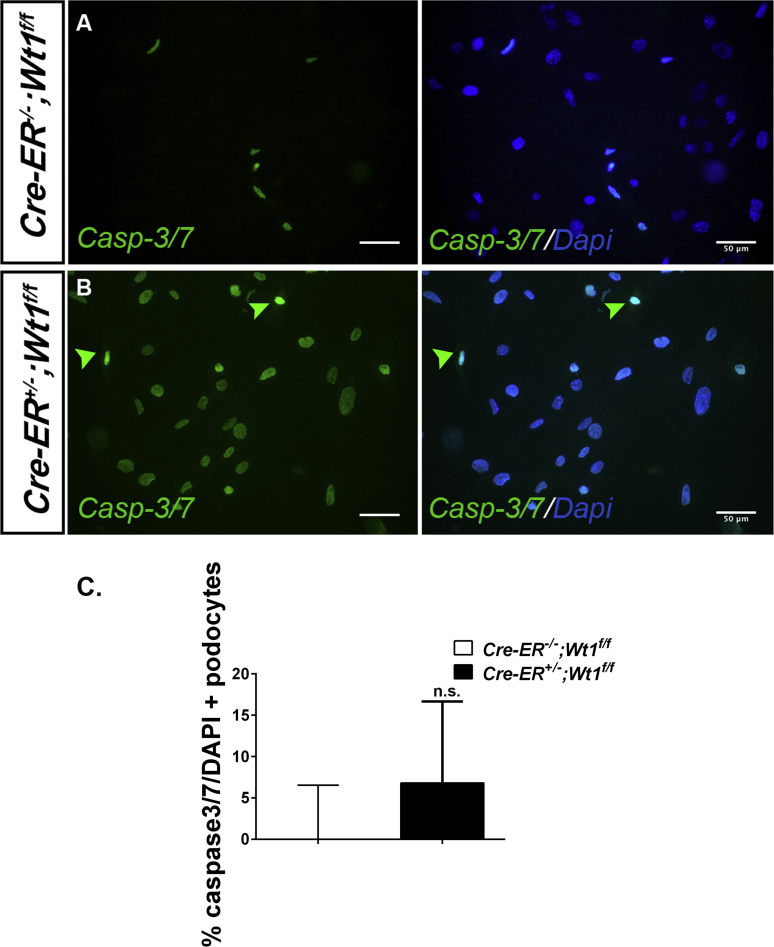
Cleaved Caspase 3/7 is induced in primary *CAGG-CreER*^*TM+/−*^*;Wt1*^*f/f*^ transgenic podocytes. (**A,B**) Representative micrographs following immunofluorescent labeling with anticleaved Caspase-3 & 7 (Alexa 488–conjugated secondary antibody, green arrows) of primary podocytes isolated at D6 P.I. from control (**A**) and mutant (**B**) mice. (**C**) Quantitative graph showing the proportion of median number of cleaved Caspase-3/7–positive podocytes in mutants and controls. Bars represent the median of each group. Error bars represent the interquartile ranges. Median percentage of DAPI-positive, cleaved Caspase 3/7–positive podocytes in controls (*n* = 9) versus mutants (*n* = 15): 0% (IQR: 0, 6.56) versus 6.8% (IQR: 0, 16.7), *P* = 0.19 (not significant), Mann-Whitney U test.

**Figure S8:**
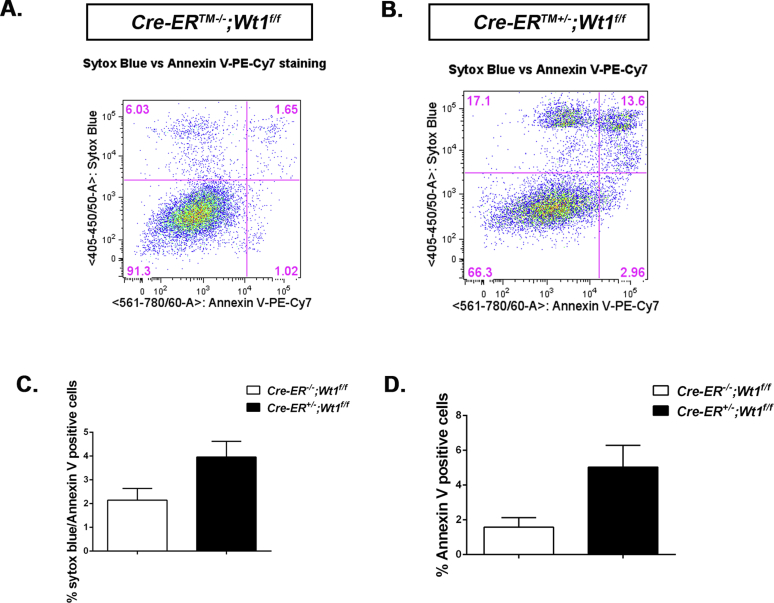
Podocyte apoptosis is increased in *CAGG-CreER*^*TM+/−*^*;Wt1*^*f/f*^ transgenic mouse podocytes at day 8 postinduction (**A-C**). (**A,B**) Representative flow cytometry results for primary podocytes from day 8 postinduction *CAGG-CreER*^*TM −/−*^*;Wt1*^*f/f*^ control and *CAGG-CreER*^*TM +/−*^*;Wt1*^*f/f*^ mutant transgenic mice. The abscissa and ordinate represent the fluorescence intensity of Annexin V Cy7 and Sytox blue, respectively. (**C**) Quantitative analysis of podocyte cell death. Bars represent the mean percentages ± SEM of Annexin V–positive/Sytox blue–positive (late apoptotic/necrotic) podocytes. *CAGG-CreER*^*TM −/−*^*;Wt1*^*f/f*^ controls (*n* = 2) versus *CAGG-CreER*^*TM +/−*^*;Wt1*^*f/f*^ mutants (*n* = 2): 2.1 ± 0.5% versus 4.0 ± 0.7%, *P* = 0.16 (not significant). Analyzed by Student *t*-test. (**D**) Annexin V/Sytox–negative cells (early apoptosis); 1.6 ± 0.56% versus 5.0 ± 1.3%, *P* = 0.13*.* Analyzed by Student *t*-test.

**Figure S9:**
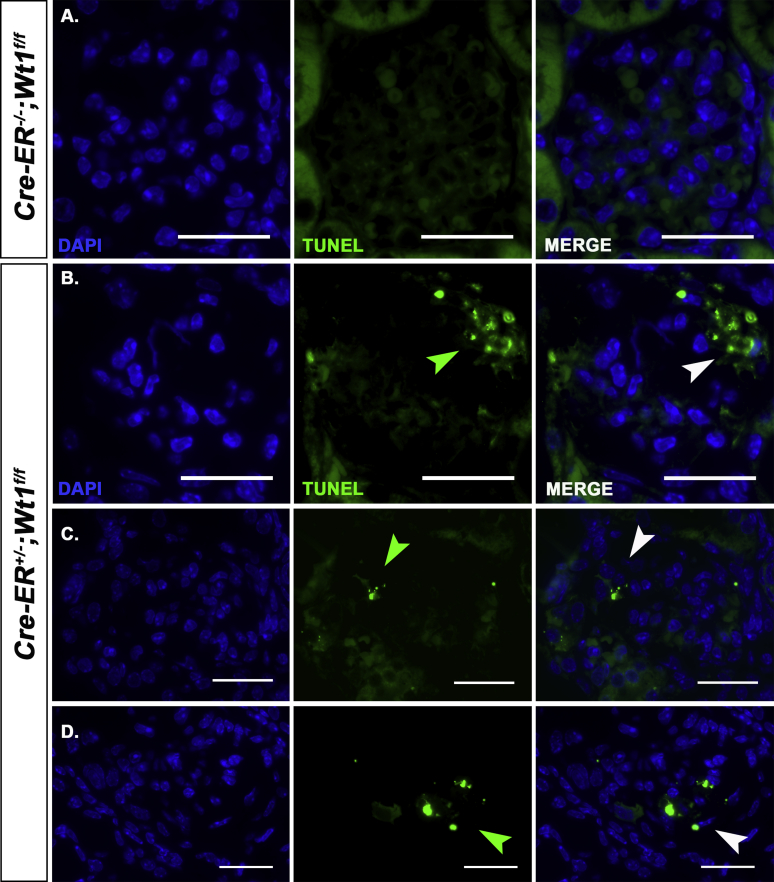
Terminal deoxynucleotidyltransferase–mediated 2′-deoxyuridine 5′-triphosphate nick end-labeling (TUNEL)-positive cells in glomeruli of *CAGG-CreER*^*TM+/−*^*;Wt1*^*f/f*^ transgenic mice at day (D) 12 postinduction (PI). (**A-D)** Shown are micrographs of glomeruli of transgenic mouse kidney tissue sections labeled with TUNEL (green arrows) and counterstained with 4′,6-diamidino-2-phenylindole (DAPI) at D12 PI. Bars = 25 μm. (**A**) *CAGG-CreER*^*TM−/−*^*;Wt1*^*f/f*^ controls display no TUNEL-positive cells in the glomerulus. (**B-D**) Representative micrographs for D12 PI *CAGG-CreER*^*TM−/−*^*;Wt1*^*f/f*^ mutants exhibiting TUNEL-DAPI-positive cells in the glomeruli. See also Figure 2c for TUNEL-positive primary podocytes of mutant versus control mice.

Podocyte apoptosis has previously been implicated in the pathogenesis of glomerulosclerosis.^30^ Therefore, we next investigated whether apoptosis is evident during disease induction in *CAGG-CreER*^*TM+/−*^*;Wt1*^*f/f*^ transgenic mice. Expression of Cleaved Caspase-3 protein was observed within mutant glomeruli as early as D4 PI but quantitatively, was not statistically significantly different compared with control glomeruli (Figure 2a) (*P =* 0.06, NS). By D5 PI, at the onset of early glomerulosclerosis, we observed an increased number of Cleaved Caspase-3/DAPI-positive cells in mutant glomeruli (Figure 2c, c′, and d) (***P < 0.0001), consistent with a temporal increase in apoptosis. Terminal deoxynucleotidyltransferase–mediated 2′-deoxyuridine 5′-triphosphate nick end-labeling (TUNEL)-positive mutant glomerular cells were evident at D5 PI but were absent in controls (Supplementary Figure S6). TUNEL staining of D6 PI primary mutant podocytes was also higher than for controls (Figure 2e, e′, and f) (*P = 0.04). At the same time point, we observed increased Caspase-3/7 positivity in primary mutant podocytes (Supplementary Figure S7) and an increased number of Annexin V/Sytox blue-positive primary mutant podocytes compared with controls (Supplementary Figure S8). TUNEL staining at D12 PI revealed clusters of TUNEL-positive cells in peripheral segments of mutant glomeruli not evident in the glomeruli of controls (Supplementary Figure S9). Together, these studies suggest that loss of *Wt1* in mature podocytes is associated with podocyte apoptosis and development of glomerulosclerosis.

### Podocyte Notch activation precedes glomerulosclerosis in *CAGG-CreER*^*TM+/−*^*;Wt1*^*f/f*^ transgenic mice

**Figure 3:**
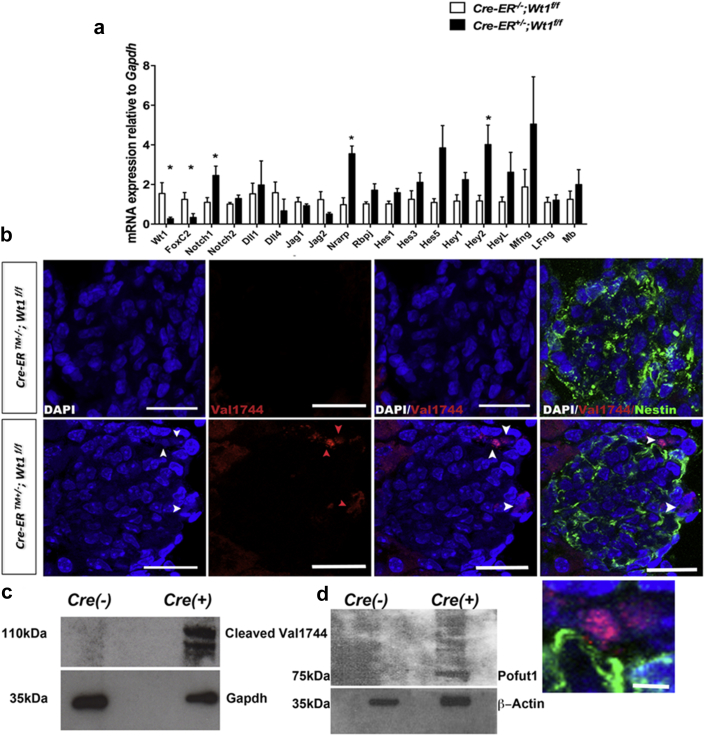
**Notch activation coincides with onset of glomerulosclerosis in in *CAGG-CreER***^***TM−/+***^***;Wt1***^***f/f***^**transgenic mice following tamoxifen administration.** (**a**) Relative mean transcript levels of Notch pathway components in primary podocytes of mutants compared with controls at day (D) 6 post-induction (PI). Bars represent means and error bars represent SDs. *CAGG-CreER*^*TM−/−*^*;Wt1*^*f/f*^ (*n* = 6) versus *CAGG-CreER*^*TM+/−*^*;Wt1*^*f/f*^ (*n* = 10): mean *Notch1*, 1.1 ± 0.2 versus 2.5 ± 0.5, *P = 0.05; mean *Nrarp,* 0.9 ± 0.4 versus 3.6 ± 0.4, **P = 0.001; mean *Rbpsuh*, 1.0 ± 0.1 versus 1.7 ± 0.3, *P* = 0.09; mean *Hes1*, 1.0 ± 0.1 versus 1.6 ± 0.2, *P* = 0.09; mean *Hes3,* 1.3 ± 0.4 versus 2.1 ± 0.5, *P* = 0.25; mean *Hes5*, 1.0 ± 0.2 versus 3.9 ± 1.1, *P =* 0.07; mean *Hey1*, 1.1 ± 0.3 versus 2.2 ± 0.4, *P* = 0.11; mean *Hey2*, 1.2 ± 0.3 versus 4.0 ± 1, *P = 0.04; mean *HeyL,* 1.1 ± 0.2 versus 2.3 ± 0.5, *P* = 0.14; mean *MFng*, 1.9 ± 0.9 versus 5.0 ± 2.4, *P* = 0.19; mean *FoxC2,* 1.2 ± 0.4 versus 0.3 ± 0.2, *P = 0.04, Student *t*-test. (**b**) Shown are representative images of glomeruli following double immunofluorescence labeling of D6 PI mouse kidney sections with anticleaved Notch1 (Val1744) and anti-Nestin of *CAGG-CreER*^*TM−/−*^*;Wt1*^*f/f*^ (controls) and *CAGG-CreER*^*TM+/−*^*;Wt1*^*f/f*^ (mutants) transgenic mice following multichannel labeling with Alexa Fluor 488–conjugated secondary antibody (Nestin, demarcates podocytes) and Alexa Fluor 594–conjugated secondary antibody (cleaved Notch1). Sections are counterstained with DAPI. Arrows show positive cleaved Notch1 nuclear staining in mutant glomeruli within Nestin-positive cells that are not evident in control glomeruli. Bars = 50 μm. Inset shows high-power view of same cells. Bar = 10 μm. (**c,d**) Representative images of Western blot analyses of protein derived from primary podocytes isolated from mutants and control mice. Immunoblots show that cleaved Notch1 (Val1744) (**c**) and Pofut1 (**d**) are expressed at D6 PI in mutant podocytes but not in controls. To optimize viewing of this image, please see the online version of this article at www.kidney-international.org.

**Figure S10:**
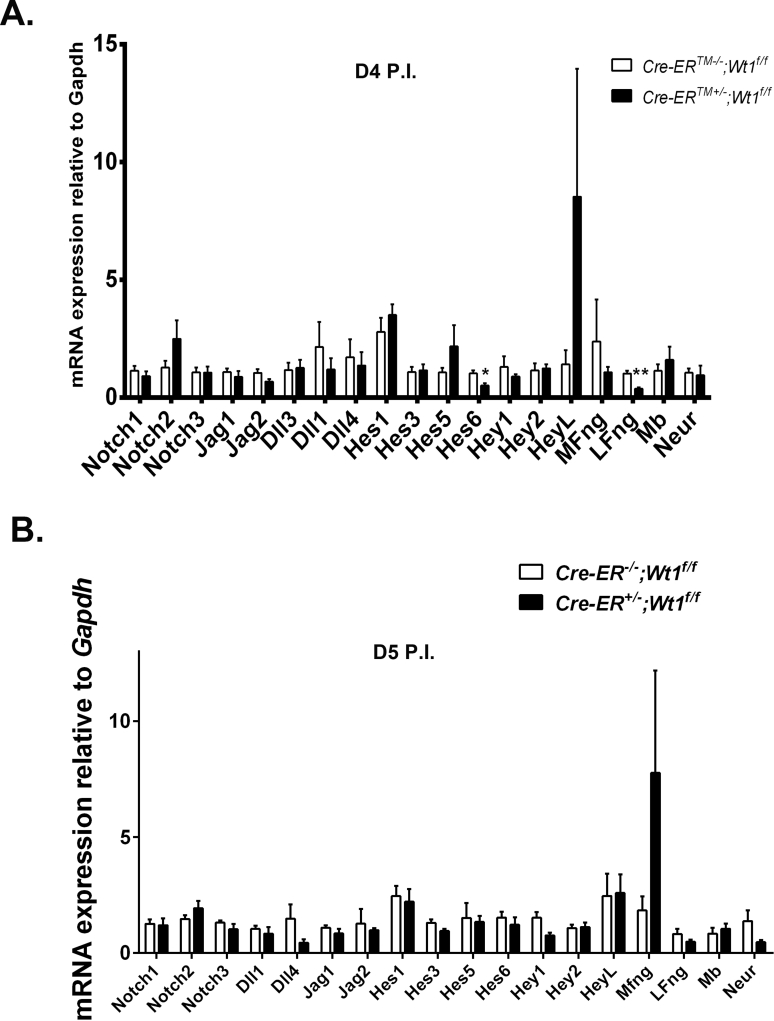
Relative transcript levels of the Notch pathway components in primary podocytes of *CreER*^*TM−/−*^*;Wt1*^*f/f*^ versus *CreER*^*TM+/−*^*;Wt1*^*f/f*^ at day (D) 4 postinduction (PI) and D5 PI. (**A**) Notch pathway transcripts in D4 PI *CreER*^*TM+/−*^*;Wt1*^*f/f*^ mutants compared with *CreER*^*TM−/−*^*;Wt1*^*f/f*^ controls. Mean mRNA transcripts relative to *Gapdh* (± SEM, *CreER*^*TM−/−*^*;Wt1*^*f/f*^ versus *CreER*^*TM+/−*^*;Wt1*^*f/f*^); *Notch1*: 1.1 ± 0.2 versus 0.9 ± 0.2 (*P* = 0.45); *Notch2*: 1.3 ± 0.3 versus 2.5 ± 0.8 (*P* = 0.18); *Hes1*: 2.8 ± 0.6 versus 3.5 ± 0.4 (*P* = 0.37); *Hes5*: 1.1 ± 0.2 versus 2.2 ± 0.9 (*P* = 0.27); *HeyL*: 1.4 ± 0.6 versus 8.5 ± 5.4 (*P* = 0.23); *Mfng*: 1.8 ± 0.6 versus 7.8 ± 4.4 (*P* = 0.16), Student *t*-test. (**B**) Notch pathway transcripts in D5 PI *CreER*^*TM+/−*^*;Wt1*^*f/f*^ mutants compared with *CreER*^*TM−/−*^*;Wt1*^*f/f*^ controls: Notch1: 1.3 ± 0.2 versus 1.2 ± 0.29 (*P* = 0.88); *Notch2:* 1.5± 0.16 versus 1.9 ± 0.31 (*P* = 1.47); *Hes1:* 2.5 ± 0.4 versus 2.2 ± 0.5 (*P* = 0.73); *Hes3:* 1.2 ± 0.1 versus 0.88 ± 0.09 (*P* = 0.05); *Hes5:* 1.5 ± 0.6 versus 1.3 ± 0.25 (*P* = 0.8); *HeyL:* 2.5 ± 0.97 versus 2.6 ± 0.8 (*P* = 0.9); *Mfng:* 1.8 ± 0.6 versus 7.8 ± 4.4 (*P* = 0.16), Student *t*-test.

Constitutive Notch activation in terminally differentiated podocytes results in podocyte apoptosis and glomerulosclerosis,^27^, ^28^ supporting a role for Notch in podocyte injury. Following our observation of increased podocyte apoptosis in *CAGG-CreER*^*TM+/−*^*;Wt1*^*f/f*^ transgenic mice, we hypothesized that podocyte Notch activation plays a role in early *Wt1* glomerulopathy. At D4 PI before manifestation of disease, Notch pathway transcripts in primary podocytes of *CAGG-CreER*^*TM+/−*^*;Wt1*^*f/f*^ transgenic mice were not statistically different to those of controls (Supplementary Figure S10). By D6 PI, concomitant with early glomerulosclerosis and albuminuria, we observed increases in canonical Notch pathway transcripts, *Notch1*, *Nrarp,* and *Hey2*. Increased levels of other Notch basic helix loop helix (bHLH) transcription factors *Hes1*, *Hes3*, *Hes5,* and *HeyL* were also observed in primary mutant podocytes but not in controls (Figure 3a), although these changes did not reach statistical significance. Using double immunofluorescence labeling, we observed cleaved Notch1 protein in nuclei of Nestin-positive podocytes as early as D4 PI in mutant kidney sections (Figure 3b and c). These findings were validated by Western blot analysis showing increased cleaved Notch1 protein in primary mutant podocytes relative to controls (Figure 3d). Increased podocyte *Manic fringe* *(MFng)* transcript was also observed in mutants compared with controls (Figure 3a). Manic Fringe is a glycosyltransferase that mediates glycosylation of the extracellular domain of Notch receptors during signal transduction.^31^, ^32^ We also found upregulation of Pofut1 protein in primary mutant podocytes at D6 PI (Figure 3e). Pofut1 is an O-fucosyltransferase 1 enzyme that mediates fucosylation of the extracellular domain of the Notch.^33^ Together, these data suggest that post-translational modification of Notch components are active in early *Wt1* glomerulopathy.

**Figure 4:**
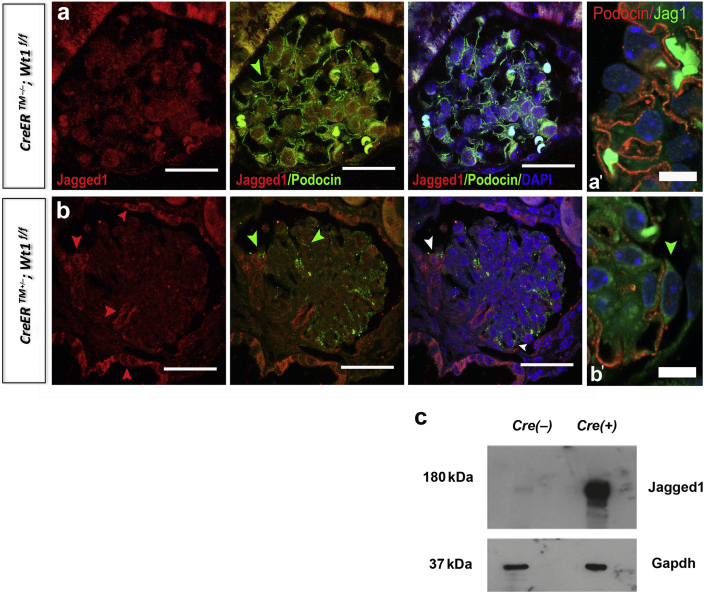
**Jagged 1 is expressed in glomeruli of *CAGG-CreER***^***TM−/+***^***;Wt1***^***f/f***^**transgenic mice at day (D) 6 postinduction (PI).** (**a,b**) Shown are representative images of glomeruli following double immunofluorescence labeling of D6 PI mouse kidney sections with anti-Jagged1 and anti-Podocin of *CAGG-CreER*^*TM−/−*^*;Wt1*^*f/f*^ (controls, **a**) and *CAGG-CreER*^*TM+/−*^*;Wt1*^*f/f*^ (mutant, **b**) mice following multichannel labeling with Alexa Fluor 488–conjugated secondary antibody (Podocin, demarcates podocytes) and Alexa Fluor 594–conjugated secondary antibody (Jagged1). Sections are counterstained with 4′,6-diamidino-2-phenylindole (DAPI). Red arrows show positive Jagged1 staining in the parietal epithelium, areas of podocyte adhesion (on the surface of Podocin-positive podocytes, green arrows, [**b**]; white arrows in merged image) to Bowman’s capsule and in the glomerular stalk of mutant glomeruli that are not evident in control glomeruli. Bar = 50 μm. (**a′,b′**) Higher-power views showing perimembranous expression of Jagged1 (green) in Podocin-positive podocytes (red) in mutants (**b′**) compared to controls (**a′**). (**c**) Representative images are shown of Western blot analyses of protein derived from primary podocytes isolated from mutants and control mice. Immunoblots show that Jagged1 is increased at D6 PI in mutant podocytes but not in controls. To optimize viewing of this image, please see the online version of this article at www.kidney-international.org.

We next sought to determine whether the Notch ligand Jagged1 is expressed in *CAGG-CreER*^*TM*^^*+/−*^*;Wt1* ^*f/f*^ transgenic mice owing to previous studies implicating podocyte Jagged1 in glomerulosclerosis.^28^ We observed a striking upregulation of Jagged1 protein in the parietal epithelium of mutants compared with controls (Figure 4a and b). Within segments of mutant glomeruli, foci of Podocin-positive cells expressing Jagged1 were also observed (Figure 4b′, high-power images). Immunoblotting of primary mutant and control podocyte lysates revealed induction of Jagged1 expression at D6 PI (Figure 4c). Together, these data suggest that Notch signaling is activated when glomerulosclerosis first manifests following deletion of *Wt1* in mature podocytes.

Given previous studies that showed that Wt1 and Foxc1a can inhibit NICD activation,^34^ we explored FoxC2 expression in this model. Using semiquantitative PCR analysis, we found decreased podocyte *FoxC2* transcript in *CAGG-CreER*^*TM+/−*^*;Wt1* ^*f/f*^ transgenic mice compared with their control littermates (*P = 0.04) (Figure 3a). This decrease was observed at the same time point when Notch components were upregulated and concordant with disease manifestation. These findings support the hypothesis that Notch activation in mature podocytes is normally repressed by FoxC2 and WT1 and loss of these proteins is associated with increased podocyte Notch activation.

### Podocyte HES1 expression is evident at onset of glomerulosclerosis and is associated with podocyte epithelial to mesenchyme gene expression

**Figure 5:**
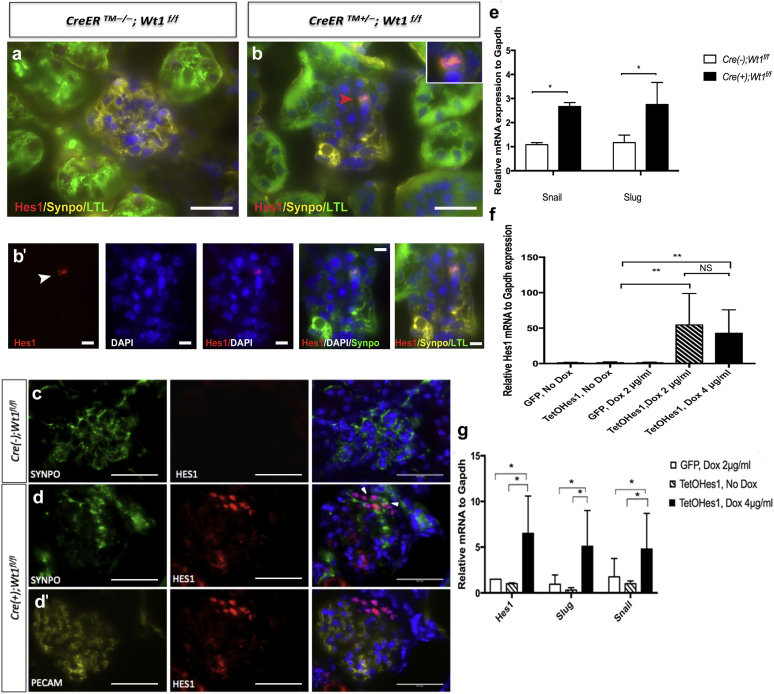
**Hairy enhancer of split 1** (**HES1) expression coincides with onset of glomerulosclerosis in *CAGG-CreER***^***TM+/−***^***;Wt1***^***f/f***^**transgenic mice.** (**a,b,b′**) Shown are representative images of glomeruli following double immunofluorescence labeling of day (D) 5 postinduction (PI) mouse kidney sections with anti-HES1, anti-Synaptopodin, and *Lotus tetragonolobus* lectin (LTL) of *CAGG-CreER*^*TM−/−*^*;Wt1*^*f/f*^ (mutant) and *CAGG-CreER*^*TM+/−*^*;Wt1*^*f/f*^ (control) transgenic mice following multichannel labeling with Alexa Fluor 488–conjugated secondary antibody (LTL, demarcates tubules), Alexa Fluor 594–conjugated secondary antibody (Hes1), and Alexa Fluor 647–conjugated secondary (Synaptopodin, demarcates podocytes). Sections are counterstained with 4′,6-diamidino-2-phenylindole (DAPI). Bars = 50 μm. (**b,b′**) In mutants, HES1-positive, Synaptopodin-positive glomerular epithelial cells were observed in regions distinct from LTL-positive tubules. Bars = 50 μm. (**b′**) Bars = 10 μm. (**c,d,d′**) Segmental clusters of nuclear HES1 expression in Synaptopodin-positive, platelet–endothelial cell adhesion molecule (PECAM)-negative podocytes are evident in mutant glomeruli and not evident in control glomeruli. Bars = 50 μm. (**e**) Upregulation of podocyte *Snail* and *Slug* transcript at onset of glomerulosclerosis at D6 PI. Median *Snail* mRNA expression at D6 PI in control versus mutant mice: 1.1 (interquartile range [IQR]: 0.9, 1.2) versus 2.7 (IQR:1.8, 2.8), *P = 0.045, Mann-Whitney test. Median *Slug* mRNA expression at D6 PI in control versus mutant mice: 1.2 (IQR: 0.6, 1.5) versus 2.8 (IQR: 1.4, 3.7), *P = 0.03, Mann-Whitney test. (**f**) Increase in *Hes1* mRNA in doxycycline-treated primary *Nphs2;rtTA* podocytes transduced with TetOHes1 plasmid compared with untreated TetOHes1 and treated green fluorescence protein (GFP)–transduced primary *Nphs2;rtTA* podocytes. Mean *Hes1* mRNA expression relative to *Gapdh* (±SD): untreated control (GFP) versus untreated TetOHes1 versus treated control (doxycycline 2 μg/ml) versus treated TetOHes1 (2 μg/ml) versus treated TetOHes1 (4 μg/ml): 1.15 ± 0.66 versus 1.26 ± 1.13 versus 1.21 ± 0.88 versus 54.56 ± 44.24 (**P < 0.004) versus 42.78 ± 33.07 (**P < 0.008). No significant difference in dose observed, *P* = .045 (not significant [NS]). (**g**) Upregulation of *Snail* and *Slug* transcripts, genes implicated in epithelial to mesenchyme transition in doxycycline-treated primary *Nphs2;rtTA* podocytes transduced with TetOHes1 plasmid compared with untreated TetOHes1 and treated GFP-transduced primary *Nphs2;rtTA* podocytes. Untreated control (GFP) versus untreated TetOHes1 versus treated TetOHes1 (4 μg/ml): mean *Snail* mRNA expression relative to *Gapdh* (±SD): 1.76 ± 2.1 versus 1.01 ± 0.31 versus 4.82 ± 3.94, *P < 0.05. Mean *Slug* mRNA expression relative to *Gapdh* (±SD): 0.95 ± 1.12 versus 0.31 ± 0.26 versus 5.10 ± 0.3.9 (*P < 0.05). To optimize viewing of this image, please see the online version of this article at www.kidney-international.org.

**Figure S11:**
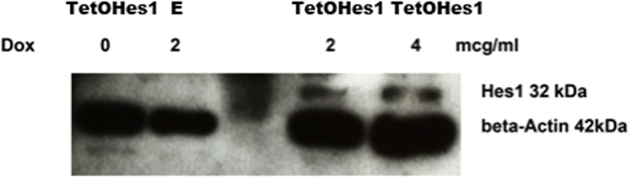
Hes1 protein expression is increased following doxycycline treatment in transduced *Nphs2;rtTA* podocytes. Representative image of a Western blot analysis of protein lysates from doxycycline-treated primary *Nphs2;rtTA* podocytes transduced with TetOHes1 and green fluorescent protein plasmids. Hes1 protein expression was increased following an increased dose of doxycycline in the TetOHes1 transduced podocytes and no Hes1 protein expression was observed in green fluorescent protein–transduced *Nphs2;rtTA* podocyte lysates postdoxycycline treatment.

Following detection of podocyte *Hes/Hey* mRNA expression in primary mutant podocytes, we sought to validate the expression of HES1, a bHLH transcription factor in podocytes by triple immunofluorescence labeling. We observed clusters of HES1-expressing cells that were positive for the podocyte marker, Synaptopodin, at onset of glomerulosclerosis in mutants compared with controls (Figure 5). HES1-positive glomerular epithelial cells were observed in regions distinct from HES1-positive *Lotus tetragonolobus* lectin (LTL)-positive tubules (Figure 5b and b′). Clusters of Synaptopodin-positive HES1-podocytes were distinct from platelet–endothelial cell adhesion molecule–positive glomerular endothelial cells (Figure 5c, d, and d′). As HES1 has been previously implicated in epithelial to mesenchymal transition (EMT), we next determined that *Snail* and *Slug* transcript was upregulated in primary D6 PI mutant podocytes compared with controls (Figure 5e). To further understand the role of HES1 in podocytes, we transfected cultured primary *Nphs2;rtTA* transgenic murine podocytes with constructs expressing either TetOHes1 or green fluorescent protein (GFP) alone. HES1 was not expressed in untreated TetOHes1 nor doxycycline-treated GFP transfected *Nphs2;rtTA* podocytes. Treatment with doxycycline led to a dose-dependent increase in podocyte *Hes1* mRNA and protein expression (Figure 5f, Supplementary Figure S11). Induction of *Hes1* expression led to a 3-fold upregulation of *Snail* and *Slug* transcript compared with untreated TetOHes1 transfected and doxycycline-treated GFP transfected *Nphs2;rtTA* podocytes (Figure 5f). These results suggest that podocyte HES1 induction could mediate manifestation of glomerulosclerosis through regulation of EMT genes in podocytes.

**Figure 6:**
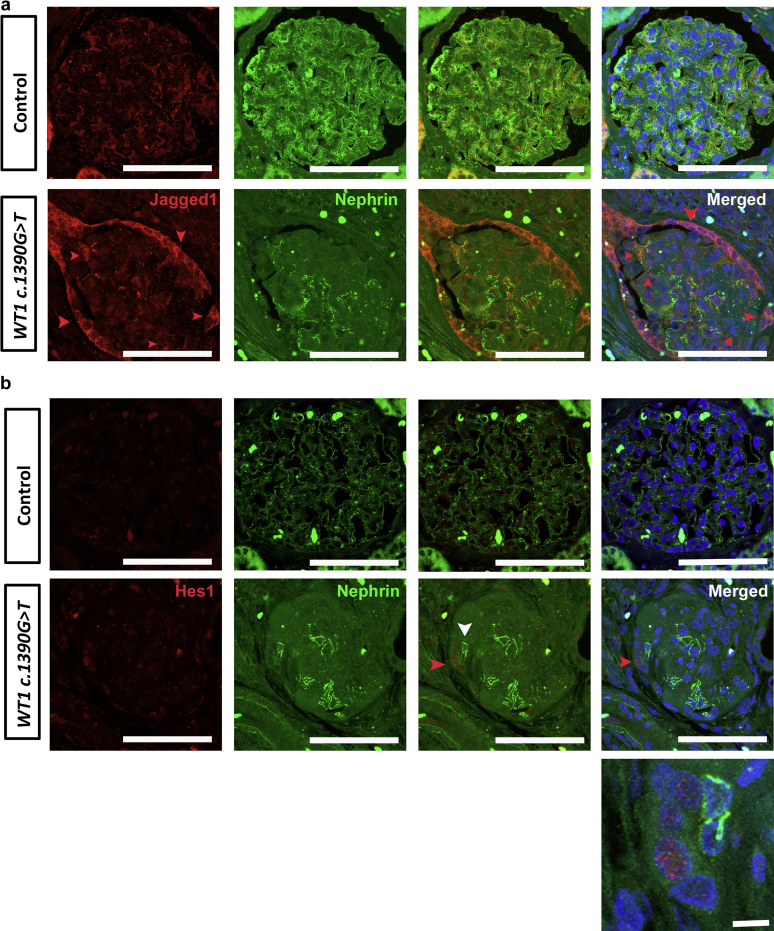
**JAGGED1 and hairy enhancer of split 1 (HES1) are expressed in Wilms’ tumor 1 (*WT1*) *c.1390G>T* mutant glomeruli.** (**a,b**) Shown are representative micrographs of glomeruli following double immunofluorescence labeling of kidney sections from control (time-zero renal allograft biopsy) and *WT1 c.1390G>T* mutant patient with anti-JAGGED1 (**a**) or anti-HES1 (**b**) antibodies (labeled with Alexa Fluor 594) and anti-Nephrin antibody (demarcates podocyte slit diaphragm, and labeled with Alexa Fluor 488). (**a**) JAGGED1 is upregulated in parietal epithelium of *WT1 c.1390G>T* mutant patient glomeruli compared with controls (red arrows) and is also expressed in a linear pattern associated with focal nephrin staining in podocytes (white arrows). JAGGED1 expression is also expressed in the endothelium of time-zero allograft glomeruli that do not exhibit glomerulosclerosis. Bars = 50 μm. (**b**) HES1, the canonical Notch basic helix loop helix transcription factor, is expressed basally in most cell types of both control and mutant glomerular tissue. Increased HES1 expression is observed in nuclei of cells adjacent to Nephrin-positive podocytes of *WT1 c.1390G>T* mutant patient glomeruli with advanced glomerulosclerosis. Bars = 50 μm. To optimize viewing of this image, please see the online version of this article at www.kidney-international.org.

Following our observation of Notch activation in murine *Wt1* glomerulopathy, we next tested biopsy samples from a human subject with FSGS associated with the *WT1 c.1390G>T* mutation. We found JAGGED1 expression in cells with focal nephrin staining but also found that expression was most marked in the parietal epithelium (Figure 6). The control biopsy from a nondiseased time 0 renal allograft biopsy revealed JAGGED1 expression in regions distal to nephrin staining suggesting endothelial JAGGED1 expression. JAGGED1 expression in the control parietal epithelium was much less than in mutant *WT1* patient tissue. Expression of the Notch bHLH transcription factor, HES1, was also observed in nuclei of mutant *WT1* glomeruli compared with in control biopsy tissue (Figure 6). We conclude that these data support a role for Notch activation in human *WT1*-mediated glomerular disease.

### Gamma secretase inhibition of Notch ameliorates disease severity in *Wt1* glomerulopathy

**Figure 7:**
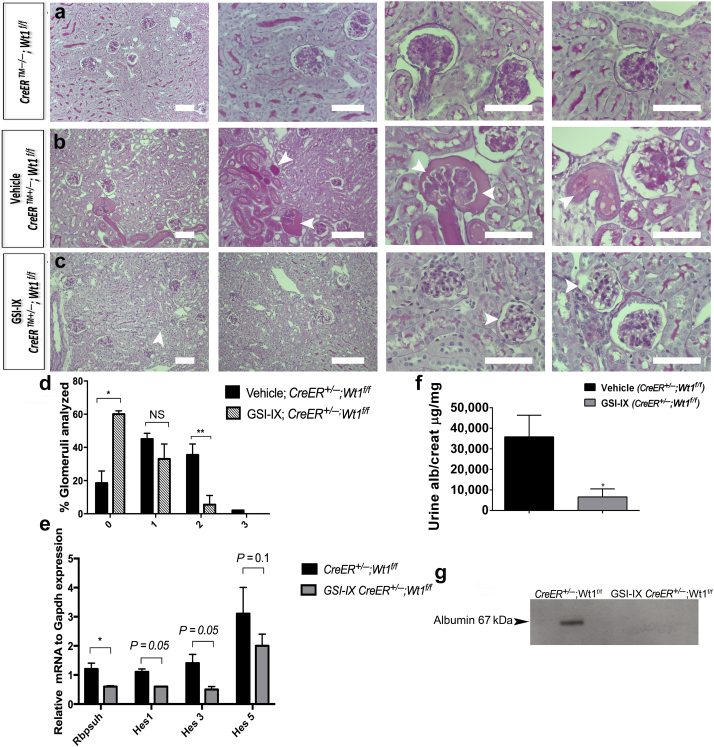
**Gamma secretase inhibition of Notch ameliorates early Wilms’ tumor 1 (*Wt1*) glomerulopathy**. (**a–c**) Shown are representative micrographs of periodic acid–Schiff–stained sections of glomeruli of *CAGG-CreER*^*TM−/−*^*;Wt1*^*f/f*^ (**a**), vehicle (dimethylsulfoxide)-treated *CAGG-CreER*^*TM+/–*^;*Wt1*^*f/f*^ (**b**), and gamma secretase inhibitor (GSI-IX)-treated *CAGG-CreER*^*TM+/−*^*;Wt1*^*f/f*^ transgenic mice (**c**) isolated late on day 5 postinduction. Bars = 50 μm. (**b**) Vehicle-treated mice exhibit glomerulosclerosis with hyaline material in the tubules that was not seen in control mice (**a**). Glomeruli of GSI-IX–treated mutants (**c**) do not show the same extent of glomerulosclerosis as vehicle-treated mutants. (**d**) Quantitative graphs of a proportion of glomeruli per genotype revealing the extent of intraglomerulosclerosis (score 0: <25% glomerulus sclerosed; score 1: 25%–50% sclerosis; score 2: 50%–75% sclerosis; score 3: >75% sclerosis). Bars represent the mean percentage of each score per genotype. Error bars represent the SEMs. At day 5 postinduction, a higher proportion of GSI-IX–treated *CAGG-CreER*^*+/−*^*;Wt1*^*f/f*^ transgenic mice (*n* = 52 glomeruli, *n* = 5 mice) exhibited normal glomerular morphology compared with vehicle-treated *CAGG-CreER*^*+/−*^*;Wt1*^*f/f*^ (mutants) (*n* = 52 glomeruli, *n* = 4 mice): vehicle-treated *CAGG-CreER*^*+/−*^*;Wt1*^*f/f*^ versus GSI-IX–treated *CAGG-CreER*^*+/−*^*;Wt1*^*f/f*^ mutant transgenic mice: score 0: 18 ± 0.9% versus 59 ± 1.0%, *P < 0.02, Student *t*-test; score 2: 39 ± 5% versus 8 ± 3%, **P < 0.008, Student *t*-test. (**e**) Quantitative graph showing reduced mean relative *Rbpsuh*, *Hes1*, *Hes3,* and *Hes5* mRNA expression in podocytes from GSI-IX–treated and untreated mutant mice: *CAGG-CreER*^*+/−*^*;Wt1*^*f/f*^ versus GSI-IX–treated *CAGG-CreER*^*+/−*^*;Wt1*^*f/f*^ mice: *Rbpsuh*: 1.2 ± 0.2 versus 0.6 ± 0.03, *P = 0.01; *Hes1*: 1.1 ± 0.1 versus 0.6 ± 0.01, *P* = 0.05; *Hes3*: 1.4 ± 0.3 versus 0.5 ± 0.1, *P* = 0.05; *Hes5*: 3.1 ± 0.9 versus 2.0 ± 0.4, *P* = 0.1. Bars represent means and error bars represent SEMs. (**f**) Quantitative graph showing median urine albumin-creatinine ratio in vehicle-treated versus GSI-IX–treated mutant mice. Bars represent the median of each group. Error bars represent the interquartile ranges (IQRs): *CAGG-CreER*^*+/−*^*;Wt1*^*f/f*^ versus GSI-IX–treated *CAGG-CreER*^*+/−*^*;Wt1*^*f/f*^ mice: 35,836 (IQR: 21,304, 46,371) versus 6657 (IQR: 1337, 10,565), *P = 0.02, Mann-Whitney U test. (**g**) Western blot analysis demonstrates albumin in urine samples of vehicle-treated versus GSI-IX–treated mutant mice (molecular weight of albumin: 66.5 kDa). To optimize viewing of this image, please see the online version of this article at www.kidney-international.org.

**Figure S12:**
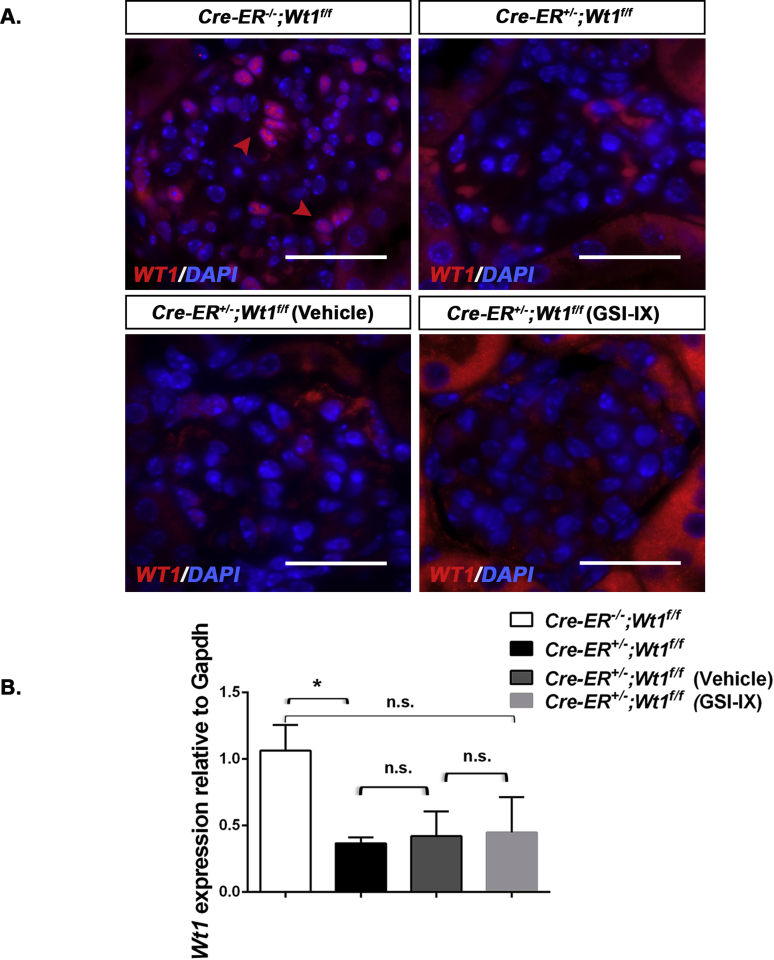
Wt1 expression is unchanged in Vehicle versus GSI-IX-treated CAGG-CreER^TM+/–^; Wt1f/f transgenic mice. (**A**) Shown are merged micrographs of kidney sections following immunofluorescent labeling of kidney tissue sections of CAGG-CreER^TM–/–^; Wt1f/f, CAGG-CreER^TM+/–^; Wt1f/f, vehicle versus GSI-IX-treated CAGG-CreER^TM+/–^; Wt1f/f transgenic mice with Wt1 (Alexa Fluor 594-conjugated secondary antibody). Scale bars = 50 μm. (**B**) Relative transcript levels of Wt1 in primary podocytes of *CAGG-CreER*^*TM−−*^*;Wt1*^*f/f*^ (control), *CAGG-CreER*^*TM+/−*^*;Wt1*^*f/f*^ (mutant), vehicle-treated *CAGG-CreER*^*TM+/−*^*;Wt1*^*f/f*^*,* and GSI-IX–treated *CAGG-CreER*^*TM+/−*^*;Wt1*^*f/f*^ transgenic mice. Bars represent the mean, error bars represent the SEM. *CAGG-CreER*^*TM−/−*^*;Wt1*^*f/f*^ versus *CAGG*-*CreER*^*TM+/−*^*;Wt1*^*f/f*^: 1.1 ± 0.2 versus 0.4 ± 0.4, *P = 0.03, Student *t*-test; *CAGG-CreER*^*TM−/−*^*;Wt1*^*f/f*^ versus vehicle-treated *CAGG-CreER*^*TM+/−*^*;Wt1*^*f/f*^ mice: 1.1 ± 0.2 versus 0.4 ± 0.2, *P* = 0.07, Student *t*-test; vehicle-treated *CAGG-CreER*^*TM+/−*^*;Wt1*^*f/f*^ mice versus GSI-IX–treated *CAGG-CreER*^*TM+/−*^*;Wt1*^*f/f*^ transgenic mice: 0.4 ± 0.2 versus 0.4 ± 0.3, *P* = 0.93, Student *t*-test. n.s., not significant.

As podocyte Notch activation is a feature of early disease, we hypothesized that pharmacological Notch inhibition at onset of glomerulosclerosis could influence severity of disease. *CAGG-CreER*^*TM+/−*^*;Wt1*^*f/f*^ transgenic mice were treated by i.p. injection with the gamma secretase inhibitor GSI-IX, N-[N-(3,5-Difluorophenacetyl)-L-alanyl]-S-phenylglycine t-butyl ester (DAPT), or dimethylsulfoxide (i.e., vehicle only) late D4 PI and early D5 PI (Figure 7a–d). In vehicle-treated mutants, we observed hyaline-filled tubules representing proteinaceous material and sclerotic glomeruli with mesangial proliferation (Figure 7b). These features were not evident in GSI-IX-treated mutants (Figure 7c). The proportion of vehicle-treated mutant glomeruli exhibiting extensive glomerulosclerosis was significantly higher than the proportion of GSI-IX-treated mutants (**P = 0.008) (Figure 7d). We validated inhibition of canonical Notch pathway transcripts by semiquantitative PCR (Figure 7e). Analysis of *Wt1* transcript and protein expression did not reveal a significant difference between vehicle-treated or GSI-IX-treated mutant mice (Supplementary Figure S12). GSI-IX-treated mutants, compared with vehicle-treated mutants, showed an improvement in urine albumin-creatinine ratio at D5 PI (Figure 7f). Western blot analysis of urine albumin confirmed absence of albuminuria in GSI-IX-treated mice compared with in vehicle-treated mutants at D5 PI (Figure 7g). We conclude that the observed efficacy of GSI inhibition in amelioration of glomerulosclerosis supports the hypothesis that Notch is activated during disease development.

**Figure S13:**
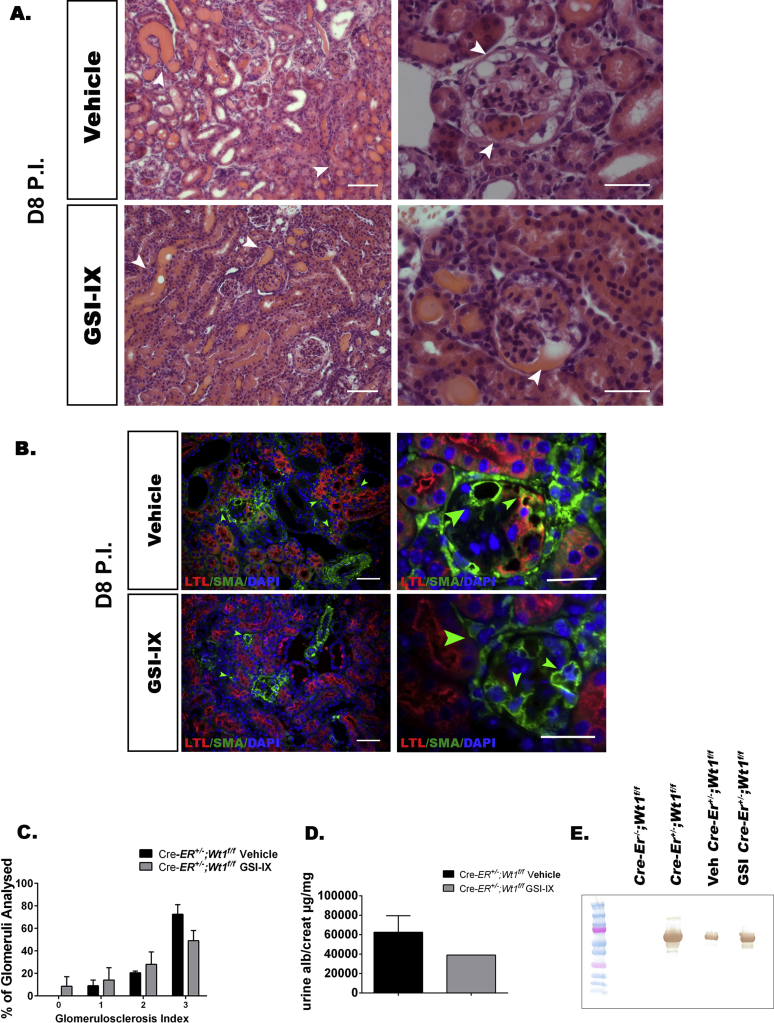
Gamma secretase inhibition (GSI-IX) in late *Wt1* glomerulopathy does not rescue disease (**A-E**). (**A**) Shown are representative micrographs of hematoxylin and eosin–stained kidney sections of GSI-IX-treated *CAGG-CreER*^*TM+/−*^*;Wt1*^*f/f*^ and vehicle (dimethylsulfoxide)-treated *CAGG-CreER*^*TM+/−*^*;Wt1*^*f/f*^ mutant mice treated the evening of day (D) 7 postinduction (PI) (established disease with renal impairment). Vehicle-treated mice (top panel) exhibit global glomerulosclerosis with hyaline material in the tubules. Top left image represents a lower-power image of the glomeruli and tubules, with hyaline material in the tubules (indicated by white arrows, bar = 50 μm); top right panel shows a high-power image of the glomerulus with global glomerulosclerosis (indicated by white arrows, bar = 25 μm). Glomeruli of GSI-IX–treated mice (bottom panel) show the same degree of glomerulosclerosis as do vehicle-treated mice, with global glomerulosclerosis. Bottom left image represents a lower-power image of the glomeruli and tubules, with hyaline-filled tubules (indicated by white arrows, bar = 50 μm); bottom right panel shows a high-power image of the glomerulus, with severe glomerulosclerosis (indicated by white arrows, bar = 25 μm). (**B**) Shown are merged micrographs of kidney sections following double immunofluorescence labeling of vehicle- and GSI-IX–treated *CAGG-CreER*^*TM+/−*^*;Wt1*^*f/f*^ kidney tissue sections with α-smooth muscle actin (SMA) (Alexa Fluor 488–conjugated secondary antibody) and anti-*Lotus tetragonolobus* lectin (LTL) (Alexa Fluor 594–conjugated secondary antibody). Bars = 50 μm. Top panel represents lower power image (left) of the tubules and glomeruli in vehicle-treated mutants, highlighting SMA between the tubules (green arrows), indicating tubulointerstitial disease; higher-power (right) shows SMA-positive staining in dilated capillary loop (green arrow). Bottom panel: low-power image (left) of the tubules and glomeruli in GSI-treated mutants, showing SMA in peritubular interstitium (green arrows); higher-power (right) image highlights the glomerulus with SMA expression within the glomerular tuft (green arrows). (**C**) Quantitative graph of glomerulosclerosis index (score 0–3: 0, <25%; 1, 25%–50%; 2, 50%–75%; 3, >75% sclerosis). Vehicle-treated mutants (*n* = 62 glomeruli, *n* = 2 mice) versus GSI-IX–treated mutants (*n* = 66 glomeruli, *n* = 2 mice). Bars represent the mean of each group, error bars represent the SEMs. Score 0: 0% versus 8.5 ± 8.5%, *P =* 0.42, Student *t*-test; score 1: 9 ± 5% versus 28.5 ± 3.5%, *P =* 0.09, Student *t*-test; score 2: 20.5 ± 1.5% versus 28 ± 11%, *P =* 0.57, Student *t*-test; score 3: 72.5 ± 8.5% versus 49 ± 9%, *P* = 0.20, Student *t*-test. (**D**) Quantitative graph showing mean urine albumin-creatinine ratio in vehicle-treated (*n* = 3) versus GSI-IX–treated (*n* = 1) *CAGG-CreER*^*TM+/−*^*;Wt1*^*f/f*^ transgenic mice. Bars represent the mean, error bars represent the SEMs. (**E**) Western blot analysis demonstrates albuminuria in D8 PI *CAGG-CreER*^*TM+/−*^*;Wt1*^*f/f*^ vehicle-treated *CAGG-CreER*^*TM+/−*^*;Wt1*^*f/f*^ and GSI-IX–treated *CAGG-CreER*^*TM+/−*^*;Wt1*^*f/f*^ transgenic mice but not in *CAGG-CreER*^*TM−/−*^*;Wt1*^*f/f*^ control mice (molecular weight of albumin: 66.5 kDa).

We also tested the role of starting treatment in established disease by administering GSI-IX to *CAGG-CreER* ^*TM+/−*^*;Wt1*^*f/f*^ mutant mice on D7 PI. Following 2 doses of GSI-IX, mice were nephrectomized late on D8 PI, and kidney histology was analyzed for 2 mice per treatment. We did not observe a difference in degree of glomerulosclerosis, albuminuria, nor tubulointerstitial expression of vascular smooth muscle actin immunofluorescence (Supplementary Figure S13). We hypothesize that podocyte Notch activation plays a role in early *Wt1* glomerulopathy.

### *Mfng* and *Rbpsuh* short hairpin RNA knockdown does not rescue podocyte EMT gene expression

**Figure S14:**
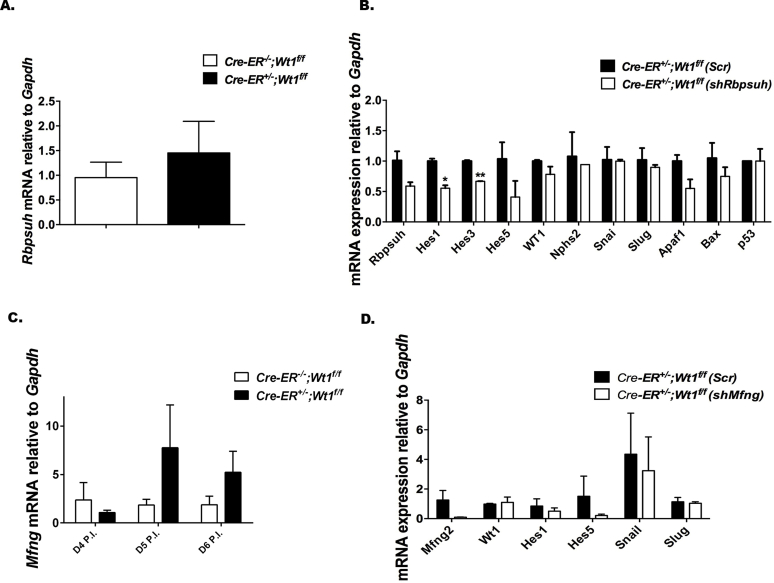
Manic fringe (Mfng) and Rbpsuh short hairpin RNA (shRNA) knockdown in day (D) 6 postinduction (PI) *CAGG-CreER*^*TM−/+*^*;Wt1*^*f/f*^ podocytes results in repression of Notch basic helix loop helix (bHLH) targets (**A–D**). (**A**) Relative transcript levels of *Rbpsuh* (canonical Notch target) in D6 PI primary podocytes of *CAGG-CreER*^*TM−/−*^*;Wt1*^*f/f*^ controls (*n* = 7) versus CAGG-CreER^TM−/+^;Wt1^f/f^ mutants (*n* = 10). Bars represent the median level, error bars represent interquartile range (IQRs). *Rbpsuh* expression is increased in D6 PI *CAGG-CreER*^*TM−/+*^*;Wt1*^*f/f*^ mutants versus *Cre-*negative controls: 0.95 (IQR: 0.79, 1.27) versus 1.45 (IQR: 1.01, 2.09), *P* = 0.05, Mann-Whitney U test. (**B**) Relative transcript levels of *Rbpsuh*, Notch bHLH components, *Hes1, Hes3,* and *Hes5, Snail, Slug* (markers of epithelial to mesenchymal transition [EMT]), podocyte-specific transcripts *Wt1*, *Nphs1,* and *Nphs2* and apoptosis components Apaf1, Bax, and p53 following 48 hours of Rbpsuh shRNA knockdown. Bars represent the mean, error bars represent the SEMs. Scrambled shRNA (Scr) *CAGG-CreER*^*TM−/+*^*;Wt1*^*f/f*^ mutants (*n* = 2) versus shRNA *Rbpsuh*-*CAGG-CreER*^*TM−/+*^*;Wt1*^*f/f*^ mutants (*n* = 2); *Rbpsuh:* 1.0 ± 0.15 versus 0.6 ± 0.1, *P* = 0.12; *Hes1*: 1.0 ± 0.04 versus 0.6 ± 0.05, *P = 0.02; *Hes3:* 1.0 ± 0.02 versus 0.7 ± 0.004, **P = 0.002; *Hes5*: 1.0 ± 0.27 versus 0.4 ± 0.3, *P* = 0.24; *Wt1*: 1.0 ± 0.02 versus 0.8 ± 0.13, *P* = 0.23; *Snail*: 1.0 ± 0.21 versus 1.0 ± 0.02, *P* = 0.92; *Slug*: 1.0 ± 0.2 versus 0.9 ± 0.04, *P* = 0.61; *Apaf1:* 1.0 ± 0.1 versus 0.71 ± 0.2, *P =* 0.31; *Bax*: 1.1 ± 0.35 versus 0.8 ± 0.2, *P* = 0.60; *p53:* 1.0 ± 0.0 versus 0.7 ± 0.2, *P* = 0.27. Student *t*-test. (**C**) Relative transcript levels of Manic fringe in *CAGG-CreER*^*TM−/−*^*;Wt1*^*f/f*^ controls versus *CAGG-CreER*^*TM−/+*^*;Wt1*^*f/f*^ mutants at D4, D5, and D6 PI. Bars represent the mean, error bars represent the SEMs. D4: *Mfng*: 2.4 ± 1.8 versus 1.1 ± 0.2, *P* = 0.44; D5: *Mfng*: 1.8 ± 0.6 versus 7.8 ± 4.4, *P* = 0.16; D6: *Mfng*: 1.9 ± 0.9 versus 5.0 ± 2.4, *P* = 0.19. Student *t*-test. (**D**) Relative transcript levels of *Rbpsuh*, Notch bHLH components, *Hes1*, *Hes3, Hes5*, *Snail*, *Slug* (markers of EMT), and podocyte-specific transcript *Wt1* following 48 hours of *MFng* shRNA knockdown. Scrambled shRNA transfected (Scr) *CAGG-CreER*^*TM−/+*^*;Wt1*^*f/f*^ (*n* = 2) versus *Manic fringe* shRNA (*shMfng*) transfected *CAGG-CreER*^*TM−/+*^*;Wt1*^*f/f*^ (*n* = 2). Bars represent the mean, error bars represent SEMs. *Mfng* expression is reduced following shRNA knockdown. *Scr* versus *shMFng*: 1.3 ± 0.7 versus 0.1 ± 0.01, *P* = 0.22. *Wt1* transcript levels are equivocal in both groups posttransfection, 1.0 ± 0.1 versus 1.1 ± 0.5, *P* = 0.78. Notch components, *Hes1,* and *Hes5* are reduced in shRNA Mfng podocytes compared with *Scr* podocytes. Mean transcript in controls versus *shRNA MFng*: *Hes1*: 0.9 ± 0.5 versus 0.5 ± 0.2, *P* = 0.58; *Hes5*: 1.5 ± 1.4 versus 0.2 ± 0.1, *P* = 0.44*.* EMT marker, Snail is reduced: 4.4 ± 2.8 versus 3.2 ± 2.3, *P* = 0.79; Slug is similar: 1.1 ± 0.3 versus 1.0 ± 0.1, *P* = 0.79. Analyzed by Student *t*-test.

Given the upregulation of *MFng* and *Rbpsuh* transcripts at D6 PI, we sought to determine whether knockdown of *Mfng* or *Rbpsuh* would influence podocyte EMT gene or apoptosis gene expression. While we did observe evidence of *Mfng* and *Rbpsuh* down-regulation with repression of *Hes1* and *Hes3* transcripts, we did not observe significant repression of podocyte *Snail* and *Slug* transcript nor significant upregulation of podocyte-specific transcripts such as *Nphs1* nor *Nphs2* (Supplementary Figure S14). Reduced primary mutant podocyte viability precluded optimal transfection owing to slow growth and increased apoptosis. We conclude that genetic rescue of *Wt1* glomerulopathy by Notch inhibition would be best validated with *in vivo* strategies.

## Discussion

Using an inducible model of *Wt1* deletion, we demonstrate a role for Notch activation in the pathogenesis of *Wt1* glomerulopathy. The data provided in this study establish the utility of temporal semiquantitative analysis of glomerular scarring as a platform for the study of early pathological events in an inducible model of mature podocyte injury. We show that podocyte apoptosis is evident as early as the fourth day following tamoxifen administration to adult *CAGG-CreER*^*TM+/−*^*;Wt1*^*f/f*^ transgenic mice before glomerulosclerosis is evident. This podocyte loss, secondary to *Wt1* deletion, increases when overt albuminuria is evident. Furthermore, we show upregulation of *Snail* and *Slug* mRNA, genes that are implicated in epithelial mesenchyme transition in primary mutant podocytes coincident with onset of glomerulosclerosis. Ectopic podocyte Notch activation in mice results in podocyte apoptosis, dedifferentiation, and both diffuse mesangial sclerosis and FSGS phenotypes,^27^, ^28^ which are also associated with mutations in *WT1.*^5^, ^7^ Following *Wt1* deletion in this model, we show upregulation of several Notch components, including *Notch1* and its transcriptional target gene, *Nrarp,*^35^ as well as Notch bHLH transcription factors. The finding of increased Jagged1 and Pofut1 protein in primary mutant podocytes at disease induction suggested a ligand-dependent mechanism of podocyte Notch activation at disease manifestation. Furthermore, induction of HES1 expression in transgenic *Nphs2;rtTA* primary podocytes led to increased *Snail* and *Slug* expression, suggesting that Notch is activating genes promoting EMT in podocytes. Notch inhibition using gamma secretase inhibitors, on the D4 and D5 following tamoxifen administration, led to a reduction in the severity of glomerulosclerosis and albuminuria. We propose a model in which loss of *Wt1* in mature podocytes induces podocyte apoptosis and EMT, which could be mediated via activation of Notch. These findings are consistent with previous reports demonstrating podocyte Notch activation in chemically induced models of glomerulosclerosis and in human biopsies of glomerular disease.^28^, ^36^, ^37^, ^38^ Increased podocyte *Hes1*, *Hes3*, *Hes5* and *Hey1*, *Hey2,* and *HeyL* have been found in streptozotocin- and puromycin aminonucleoside–induced glomerulosclerosis.^28^ Podocyte apoptosis has been shown to play an instigating role in the pathogenesis of FSGS, and Notch activation in mature podocytes induces apoptosis.^28^, ^30^ Conditional activation of the intracellular domain of *Notch1* in mature podocytes is associated with positive TUNEL staining and upregulation of podocyte *Trp53* and *Apaf1*. Pifithrin-α inhibits apoptosis in podocytes transduced with the Notch1 intracellular domain, thereby suggesting that Notch1 induces podocyte apoptosis via the p53 pathway.^28^ Conditional deletion of podocyte *Rbpj* in mice with diabetic nephropathy is associated with reduced podocyte apoptosis providing further support for a role for Notch in podocyte apoptosis.^28^ In the current study, we speculate a role for p53-mediated apoptosis in the pathogenesis of *Wt1* glomerulopathy in our model. It would be interesting to explore whether apoptosis is inhibited with conditional inactivation of *Notch1* in podocytes of adult *CAGG-CreER*^*TM+/−*^*;Wt1*^*f/f*^ transgenic mice. Indeed, conditional deletion of *Notch1*, but not *Notch2*, in podocytes of mice with diabetic nephropathy abrogates glomerulosclerosis.^37^ Furthermore, reduced expression of primary podocyte Snail1 protein and mRNA has been observed in these mice,^37^ suggesting that podocyte *Notch1* activation is associated with both apoptosis and epithelial mesenchymal transition. Our study adds to previous studies demonstrating that ectopic Notch activation in mature podocytes is associated with the development of glomerular scarring. Conditional inactivation of *Notch1* and its transcriptional targets in both early and late stages of *Wt1* glomerulopathy would further define the role and window for Notch activation in disease pathogenesis.

Pharmacological block of Notch signaling has previously defined a narrow window for Notch in proximal nephron identity.^21^, ^22^, ^23^ However, a recent study suggests that Notch is required for the formation of all nephron segments and primes nephron progenitors for differentiation.^24^ Notch1 and Notch2 are expressed in overlapping patterns in the proximal domain of the S-shaped body where podocyte precursors reside.^23^ During terminal podocyte differentiation, *Hes*/*Hey* genes are progressively down-regulated.^25^, ^26^ Regulation of vertebrate podocyte differentiation has been proposed to involve a multimeric transcriptional network involving Wt1, FoxC1/C2, and Rbpj.^34^, ^39^ FOX transcription factor binding motifs have been found in a large proportion of WT1-bound regions supporting coordinated action of these transcription factors in regulating podocyte-specific genes.^17^, ^18^ Double knockdown of either *wt1a/rbpj* or *wt1a/foxc1a* in zebrafish caused a reduction in podocyte number in contrast to a single knockdown of any of the 3 genes, supporting a genetic interaction between these transcription factors in regulation of podocyte specification.^34^ Co-immunoprecipitation studies revealed putative interactions among Wt1, Rbpj, and FoxC2 proteins, and together, combinations of Wt1, FoxC1/2, and NICD can synergistically induce *Hey1* expression.^34^ In Xenopus, knockdown of *xWT1* decreased the early glomus-specific expression of *XHRT1*, the *Hey1* orthologue, but did not perturb its late expression in the pronephros anlagen, suggesting that *xWT1* mediates expression of *XHRT1* early in glomerulogenesis.^40^ *HeyL* expression in pretubular aggregates is also regulated by Wt1 during murine metanephric development and studies of Wt1 target genes in embryonic mouse kidney tissue revealed that Wt1 can bind to the *HeyL* promoter.^41^ These studies support a role for modulation of Notch transcriptional targets by Wt1 and FoxC1/2 during nephrogenesis.

After the proximal nephron forms, podocytes function normally in the absence of Notch.^25^, ^26^ Following *Wt1* deletion in mature podocytes, we find upregulation of Notch pathway components, *Notch1*, *Nrarp*, *Hey1, Hey2, HeyL*, *Hes1*, *Hes3,* and *Hes5*. These findings are consistent with previous studies where Wt1 and Foxc1a can inhibit the ability of NICD1 to activate a synthetic Notch reporter driven by Rbpj sites^34^ and suggest a model where Wt1 and FoxC1/2 have antagonistic effects on Notch signaling in the mature podocyte. Our study also demonstrated that loss of *Wt1* in mature podocytes is associated with a reduction of *FoxC2* expression and upregulation of Notch pathway components coincident with onset of glomerulosclerosis and albuminuria. We speculate that FoxC2 could repress expression of *Hey2* and other Notch bHLH genes in mature podocytes, based on our observation of increased *Hey2* transcript in mutant podocytes at a point when *FoxC2* is down-regulated. This would be consistent with a previous report showing that *Hey2* is a transcriptional target of FoxC2 in endothelial cells.^42^ Restoration of FoxC2 levels in adult *CAGG-CreER*^*TM+/−*^*;Wt1*^*f/f*^ transgenic mice may be sufficient to restore podocyte-specific gene expression following injury, perhaps via repression of Notch bHLH gene expression.

An alternative mechanism of podocyte Notch activation in Wt1-mediated injury could also be mediated via activation of Hippo signaling. Kann *et al.*^18^ identified podocyte-specific enrichment for TEAD transcription factor motifs (effectors of Hippo signaling) in the vicinity of WT1 chromatin immunoprecipitation sequencing peaks. Notch ligands are transcriptional targets of Hippo signaling,^43^ and it is possible that loss of *Wt1* expression in mature podocytes mediates Notch activation via regulation of Hippo components.

Our observation of increased Pofut1 in mutant podocytes supports activation of Notch in *CAGG-CreER*^*TM+/−*^*;Wt1*^*f/f*^ transgenic mice. Notch activation relies on O-linked glycosylation of the extracellular domain of Notch receptors and ligands that influence receptor sensitivity to ligand stimulation.^24^ Pofut1 is an O-fucosyltransferase 1 enzyme that mediates fucosylation of the extracellular domain of the Notch protein and has recently been shown to play an important role in the regulation of cell surface expression of Notch1.^33^ We also found increased *Mfng*, a β3-*N*-acetylglucosaminyltransferase that mediates glycosylation of the Notch extracellular domain in mutant podocytes.^31^, ^32^ In most contexts, Fringe-mediated glycosylation of Notch1 renders it more sensitive to Dll1-mediated activation.^32^ We have found evidence for Jagged1 expression in *CAGG-CreER*^*TM+/−*^*;Wt1*^*f/f*^ transgenic mice and in human biopsies of *WT1*-mutated glomerulosclerosis. It will be of interest to study the consequences of *Mfng* deletion in *CAGG-CreER*^*TM+/−*^*;Wt1*^*f/f*^ mice on disease manifestation.

In the early stages of disease, we observed Jagged1 expression in podocytes and parietal epithelium of mutant glomeruli. This observation is consistent with previous reports for a role for Jagged1 in glomerulosclerosis.^28^, ^36^ In contrast, we did not observe any quantitative difference in Delta1 protein expression in primary mutant podocytes compared with controls in the early stages of disease. Both Delta1 and Jagged1 share overlapping expression patterns within the middle segment of the S-shaped body during kidney development.^23^ Combined loss of both ligands in *Six2-Cre;Dll1*^*f/f*^*;Jag1*^*f/f*^ transgenic mice results in a severe reduction in numbers of proximal tubules and glomeruli, thereby supporting a role for ligand-mediated Notch activation in defining proximal nephron identity.^23^ Replacement of one *Jag1* allele in the *Dll1*-null background can rescue some WT1-positive podocytes, suggesting that an important role for *Jag1* in podocyte fate induction.^23^ In the context of podocyte injury, Niranjan *et al.*^28^ reported that TGF-β1 mediated treatment of podocytes resulted in Jagged1 upregulation. Future studies will be directed at examining the effects of conditional deletion of *Jagged1* in adult *CAGG-CreER*^*TM+/−*^*;Wt1*^*f/f*^ mice on disease manifestation.

In summary, we identify podocyte apoptosis as an early event in the pathogenesis of glomerulosclerosis mediated by loss of *Wt1* function in mature podocytes. At disease onset, we find induction of podocyte EMT gene expression and upregulation of several Notch pathway components. Induction of podocyte HES1 expression is associated with increased *Snail* and *Slug* expression, suggesting that HES1 regulates podocyte EMT. Early pharmacological blockade of Notch signaling leads to a reduction in the severity of glomerulosclerosis and albuminuria. We speculate that activation of Notch is mediated by repression of *FoxC2*. Given the recent advances in our understanding of the complex biological roles of WT1, transgenic mice carrying point mutations relevant to human disease will provide invaluable tools to investigate the transcriptional networks and posttranscriptional mechanisms underlying WT1-related glomerulosclerosis.

## Materials and Methods

### Mouse strains

*Wt1* deletion in adult mice was achieved following generation of bitransgenic mice by crossing *CAGG* promoter–driven *CreER^TM^* mice with homozygous *Wt1* conditional mice, where the first exon of Wt1 is flanked by LoxP sites.^29^ Site-specific recombination between the LoxP sites of the *Wt1* gene results in a ubiquitous *Wt1* null allele. Successful *Wt1* deletion was demonstrated by recombination PCR and the depletion of *Wt1* expression in podocytes (Supplementary Figure S1). Mice were maintained on a mixed genetic background consisting of C57BL/6J and CD1. However, for comparison of phenotypes such as proteinuria, littermates were used.

Cre recombinase was induced by i.p. administration of tamoxifen (1 mg/40 g body weight for 3 days; Sigma Aldrich) to 5-week-old mice. All animal work was carried out under the permission of license. Mice were housed and bred in animal facilities at the Western Labs, UCL Great Ormond Street Institute of Child Health. Mice were killed at D4, D5, D6, D8, and D12 PI of tamoxifen. Bilateral nephrectomies were performed under sterile conditions.

*Nphs2;rtTA* transgenic mice expressing the tetracycline transactivator specifically in podocytes were used for primary podocyte cultures and Hes1 overexpression experiments.^44^

### Phenotype analysis

Urinary albumin and creatinine were determined using the mouse albumin enzyme-linked immunosorbent quantitation set (E90-134; Bethyl Laboratories) and creatinine assay (KGE005; R&D Systems) kits, respectively.

Western blot detection of albuminuria was determined following loading 4 microliters of urine samples (albumin antibody [E90-134; Bethyl Laboratories] 1:1000 dilution). Kidneys were fixed in in 4% formaldehyde in phosphate-buffered saline (PBS) and paraffin-embedded kidney sections were stained with PAS.

### Primary glomerular harvest and podocyte culture

Fresh kidney cortices were dissected into ice-cold Hanks balance salt solution (24020-133; Gibco/Invitrogen, Thermo Fisher Scientific), decapsulated, and minced. After rinsing thoroughly with fresh Hanks balance salt solution, the pieces were pushed through a 100-μm cell strainer (Falcon 352360; BD Biosciences) into a chilled beaker. Hanks balance salt solution was added to a total of 6 ml, and this was divided between 2 chilled 15-ml Falcon tubes. Then 2.2 ml of Percoll (17-0891-02; Amersham) was added to each tube. The samples were spun at 400 rpm for 10 minutes at 4^o^C to separate glomeruli across the Percoll gradient.^45^ The presence of glomeruli in the top of the gradient was verified microscopically. The top 1 to 2 ml were removed and passed again through a 100-μm cell strainer to catch any large tubule fragments. The filtrate was then passed through a 40-μm cell strainer (Falcon 352340, BD Biosciences) to trap the glomeruli.

For podocyte culture, harvested glomeruli were placed onto culture dishes coated with 0.1 mg/ml rat tail collagen type I as previously described. Culture medium from D1 to D3 was RPMI 1640 medium containing 15% fetal bovine serum (FBS) (Atlanta Biologicals), penicillin streptomycin, and amphotericin B. On D3 of culture, unattached glomeruli were washed away and medium was changed to 10% FBS. Podocytes were examined on D6 of culture after harvest.

### Immunofluorescence studies

Tissues were fixed and embedded in paraffin or O.C.T. Compound (Sakura Finetek) as previously described.^27^ For double and triple immunofluorescence labeling, formalin-fixed sections were deparafinized according to previously published protocols.^27^ Microwave antigen retrieval was carried out in citrate buffer in 4 5-minute cycles at medium-high setting (NN-S758WC, 950W maximum output; Panasonic) following by a 20-minute cooling period at room temperature (RT). Blocking was performed in Universal Blocking Reagent (DAKO). O.C.T. Compound (Sakura) embedded cryosections were permeabilized in 0.5% Triton X-100 for 5 minutes, followed by 2 5-minute PBS washes. They were then incubated in blocking buffer (10% goat serum, 1% BSA, 0.1% Triton X-100) for 1 hour at RT. Sections were probed with antibodies diluted in blocking reagent and incubated at 4°C overnight. The following day, sections were washed in PBS and incubated with Alexa Fluor (Molecular Probes, Invitrogen, Thermo Fisher Scientific)–conjugated secondary antibodies for 1 hour at RT. Slides were mounted using VECTASHIELD (Vector Laboratories) and nuclei were stained with DAPI. Confocal imaging was performed using a Zeiss LSM-710 system with an upright DM6000 compound microscope (Leica Microsystems) and images were processed with Zen software suite (Zen Software). Z stacks were acquired at 0.5-μm intervals and converted to single planes by maximum projection with FiJi software.

### Apoptosis studies

#### TUNEL assay

Cells were seeded on Matrigel (356234; BD Biosciences, San Jose, CA) coated 8-well chamber slides. The following day, the cells were fixed in 4% paraformaldehyde for 15 minutes and washed in PBS twice for 5 minutes each time at RT. Cells were then permeabilized with 0.2% Triton X-100 in PBS for 5 minutes, then washed in PBS for 5 minutes at RT. Then 100 μl of Equilibration buffer from the DeadEnd Fluorometric TUNEL kit (CAT.G3250; Promega) was added to the cells for 5 to 10 minutes at RT. Following Equilibration buffer, 50 μl rTdT incubation buffer (45 μl Equilibration buffer + 5 μl Nucleotide mix + 1 μl rTdT enzyme) was added to the cells; to ensure even distribution of the buffer, the slides were covered with plastic cover slips and incubated at 37°C for 1 hour. Plastic coverslips were removed by immersing the slides 2x SSC for 15 minutes at RT. Slides were then washed 3 times for 5 minutes each with PBS at RT. Sections were mounted with VECTASHIELD plus DAPI mounting medium (H-1200; Vector Laboratories). Slides were imaged on a Zeiss fluorescent microscope.

#### Cleaved Caspase-3/7 staining

Collagen-coated gas-permeable bottom plates (Corning, Corning, NY) were used to culture glomeruli for all apoptosis assays. Glomeruli were harvested from mice 6 days following tamoxifen induction. Following 6 days of glomerular harvest, unattached glomeruli were washed away with Dulbecco PBS and the medium was replaced with Roswell Park Memorial Institute with 0.2% FBS. After 18 hours, CellEvent Caspase-3/7 Green (Life Technologies, Thermo Fisher Scientific) to measure apoptosis were added to each plate per the manufacturers’ instructions. Sections were counterstained with DAPI.

#### Annexin V staining

Following tissue dissociation, the samples were spun down at 320*g* for 5 minutes and washed with the Annexin V Binding Buffer (88-8103-74; Thermo Fisher Scientific). The cells were then stained with Annexin V-PE-Cy7 for 30 minutes on ice, in the dark. After the incubation period, the samples were again washed and resuspended in the Annexin V Binding Buffer. Just prior to sample acquisition, each sample was stained with Sytox Blue at a final concentration of 0.3 mmol/l (S34857; Thermo Fisher Scientific). The samples were acquired using a 5-laser BD LSRFortessa X-20 Analyser, equipped with 355 nm (ultraviolet), 405 nm (violet), 488 nm (blue), 561 nm (yellow), and 640 nm (red) lasers. Prior to cell acquisition, the cells were filtered through a 35-mm cell strainer to prevent cellular aggregation during sample acquisition.

### Antibodies and reagents

The following antibodies were used: cleaved Notch1 (Val1744, rabbit; Cell Signaling Technologies, Danvers, MA) 1:100 immunohistochemistry, 1:50 immunofluorescence; Notch1 (D1E11; Cell Signaling) 1:1,000 Western blot; cleaved Notch2 (cleaved-Ala1734, rabbit; Sigma-Aldrich) 1:150 immunohistochemistry; cleaved Notch2 (07-1234, rabbit; Millipore) 1:200 immunofluorescence, 1:1,000 Western blot; Jagged 1(C-20, goat; Santa Cruz Biotechnology), 1:100 immunofluorescence; Podoplanin (811, hamster; Novus Biologicals, Littleton, CO), 1:100 immunofluorescence; CD31 (0026, rat; BD Pharmingen, BD Biosciences), 1:100; Hes1 (rabbit, kind gift from Professor Ryoichiro Kageyama, University of Kyoto, Japan), 1:1000; Synaptopodin (G1D4, mouse; PROGEN Biotechnik, Heidelberg, Germany); Podocin (P0372; Sigma Aldrich) 1;100; biotinylated *Lotus tetragonolobus* lectin, LTL (B-1325; Vector Laboratories) 1:100; and cleaved Caspase-3 (Asp175, rabbit; Cell Signaling Technologies), 1:400 immunofluorescence. Alexa Fluor–conjugated secondaries 488, 595, and 647 (Molecular Probes, Invitrogen, Thermo Fisher Scientific).

GSI-IX (*N*-[*N*-(3,5-difluorophen-acetyl-*L*-alanyl)]-*S*-phenylglycine *t*-butyl ester, or DAPT) were purchased from Sigma Aldrich (D5942) and administered by i.p. injection (100 μg GSI-IX/40 g body weight) on the evening of day D4 PI of tamoxifen to *CAGG-CreER*^*TM +/−*^*;Wt1*^*f/f*^ transgenic mice. Vehicle treatment with dimethyl sulfoxide (W387520; Sigma Aldrich) administered by i.p. injection (100 μg GSI-IX/40 g body weight) on the evening of D4 PI of tamoxifen to *CAGG-CreER*^*TM +/−*^*;Wt1*^*f/f*^ transgenic mice were used as controls. For late treatments, *CAGG-CreER*^*TM+/−*^*;Wt1*^*f/f*^ mutants were treated by i.p. injection with the gamma secretase inhibitor GSI-IX DAPT (100 μg/40 g mouse body weight) on the evening of D7 PI of tamoxifen and treated again with DAPT the next morning (16 hours later). Urine was collected at least 8 hours following the second DAPT treatment and mice were then sacrificed. Following nephrectomy, light microscopic examination of PAS-stained specimens were scored for severity of glomerulosclerosis. Comparison was made between GSI-DAPT and vehicle-treated *CAGG-CreER*^*TM+/−*^*;Wt1*^*f/f*^ transgenic mice.

### Transfection experiments

Primary transgenic murine *Nphs2;rtTA* podocytes were transfected with TetOHes1 plasmid (6154; Addgene)^46^ or control-GFP-only plasmid with Lipofectamine 3000 kit (L3000001; Thermo Fischer Scientific). Cells were transfected at 70% confluency. Following 24 hours, both TetOHes1 and control-plasmid transfected cells were treated with doxycycline (2 and 4 μg/ml) for 72 and 96 hours’ duration. RNA extraction was undertaken according to manufacturer’s instructions using the Qiagen microRNA extraction kit. Protein was extracted as previously described.^47^

### shRNA experiments

For *MFng* and *Rbpsuh* gene knockdown, the Thermo Scientific Open Biosystems pGIPZ MFng (V3LMM_20363), and Rbpsuh (V3LMM_437682) and nonsilencing control vectors (RHS4346) were used. Primary podocytes from D6 PI *CAGG-CreER*^*TM+/−*^*;Wt1*^*f/f*^ transgenic mice were cultured in Roswell Park Memorial Institute medium in 10% FBS and 1% insulin, transferrin, and selenium. Next, 1 × 10^5^ cells were seeded per well in 6-well dishes (plastic bottom) in duplicates, 24 hours prior to being transfected with 4 μg of *MFng* and *Rbpsuh* short hairpin RNA (shRNA) and 4 μg nonsilencing control vector. One plate was harvested to determine knockdown efficiency by quantitative real-time (qRT)-PCR 48 hours following transfection.

### RNA extraction and qRT-PCR

Following primary podocyte derivation, total RNA was isolated using the RNeasy Micro Kit (Qiagen). cDNA was synthesized by reverse transcription using a high-capacity RNA-to-cDNA kit (4387406; Life Technologies, Thermo Fisher Scientific), qRT-PCR was performed with 250 ng cDNA on a Bio-Rad qPCR machine (CFX96 touch real-time PCR detection system; Hercules, CA) using SYBR Green PCR Master Mix (Applied Biosystems) and 0.45 μg of the oligonucleotides (Sigma Aldrich) outlined in Supplementary Tables S1 and S2. For each gene, the reaction was run in duplicate for between 6 and 10 samples, and for each primer pair, a no-template control was included. The data were normalized to *Gapdh* gene levels within each sample and analyzed using the ^ΔΔ^Ct method.^48^

### Western blotting

Protein was isolated using radioimmunoprecipitation assay buffer supplemented with phosphatase and protease inhibitors. Lysis was completed by shearing through a 26-gauge syringe needle. Samples were denatured with 10% b-mercaptoethanol (Sigma Aldrich) in 4X Laemmli sample buffer at 95°C for 10 minutes. Primary podocytes were run on 4% to 15% sodium dodecylsulfate–polyacrylamide gel electrophoresis gradient gels (Bio-Rad Laboratories). Immortalized podocytes were run on 10% sodium dodecylsulfate–polyacrylamide gel electrophoresis gels. All gels were transferred onto polyvinylidene fluoride and blocked in 5% nonfat milk in PBS before being probed with primary antibodies and secondary antibodies. Blots were developed with Pierce ECL Western Blotting Substrate (Thermo Fisher Scientific).

### Statistics

Statistical analyses were performed in GraphPad Prism V.7 (GraphPad Software). For each analysis, we examined at least 6 to 10 independent samples per experimental group; for qRT-PCR analysis, the average of duplicate reactions was used as the value of that sample. Where data is normally distributed, results are expressed as mean ± SD or SEM relative to the specified controls. Where data is not normally distributed, results are reported as medians with respective interquartile ranges. For all statistical analyses, we used a 2-tailed, unpaired Student *t*-test or Mann-Whitney U test to analyze the difference between 2 groups. The Bonferroni correction was used when more >2 groups were present. Values were regarded significant if <0.05; all error bars represent SDs.

### Study approval

Animal experiments were conducted with ethical approval from the Animal Welfare and Ethical Review Body of the University College of London, Great Ormond Street Institute of Child Health and carried out under United Kingdom Home Office license 70/7892. Approval for research on human tissue was obtained from the NHS National Research Ethics Service (NRES) Committee, North-East York, UK.

## Disclosure

PJS receives lecture fees from Natera Inc. All the other authors declared no competing interests.

## Supplementary Material

Table S1 Genomic primers for genotyping.

Table S2 Real-time quantitative polymerase chain reaction primers.
