# Supplementary material for: Activation of podocyte Notch mediates early Wt1 glomerulopathy
Source: Kidney Int. 2018 Apr;93(4):903–20. doi: 10.1016/j.kint.2017.11.014 (PMC6169130; doi:10.1016/j.kint.2017.11.014)
Supplement: Table S1 — Genomic primers for genotyping. [file mmc1.docx]

**Supplementary Table S1**

**Genomic Primers for genotyping:**

| Gene | Primer sequence (5’-3’) |
| --- | --- |
| Wt1/loxp F | TGGGTTCCAACCGTACCAAAGA |
| Wt1/loxp R | GGGCTTATCTCCTCCCATGT |
| Cre F | GCATTACCGGTCGATGCAACGAGTGATGAG |
| Cre R | GAGTGAACGAACCTGGTCGAAATCAGTGCG |
| Nphs2;rtTA F | CGCACTTCAGTTACTTCAGGTCCTC |
| Nphs2;rtTA R | GCTTATGCCTGATGTTGATGATGC |
