# Supplementary material for: Activation of podocyte Notch mediates early Wt1 glomerulopathy
Source: Kidney Int. 2018 Apr;93(4):903–20. doi: 10.1016/j.kint.2017.11.014 (PMC6169130; doi:10.1016/j.kint.2017.11.014)
Supplement: Table S2 — Real-time quantitative polymerase chain reaction primers. [file mmc2.docx]

**Supplementary Table S2**

**Real-time qPCR primers.**

| Gene | Primer sequence (5’-3’) |
| --- | --- |
| Wt1 F | GGTGGCACAGTTGTCAGAAA |
| Wt1 R | GGTGAGTGGGAGGAATTTCA |
| Nphs1 F | GCCAATCAATGACAGGAGGT |
| Nphs1 R | GTCAGGTTGTTGGGCTTGTT |
| Nphs2 F | AGGGATGACAAGAAGGCAAA |
| Nphs2 R | ACCTCGTCCACGTCCACTAC |
| Notch1 F | CCAGCAGATGATCTTCCCGTAC |
| Notch1 R | TAGACAATGGAGCCACGGATGT |
| Notch2 F | TCTATCCCCCGTCGATTCG |
| Notch2 R | GATGTGATCATGGGAGAGGATGT |
| Notch3 F | CCAGGGAATTTCAGGTGCAT |
| Notch3 R | GCCGTCGAGGCAAGAACA |
| Jag1 F | ACACAGGGATTGCCCACTTC |
| Jag1 R | AGCCAAAGCCATAGTAGTGGTCAT |
| Jag2 F | CGACTCACACTGCGCTTCA |
| Jag2 R | TCGGATTCCAGAGCAGATAGC |
| Dll1 F | CATGAACAACCTAGCCAATTGC |
| Dll1 R | GCCCCAATGATGCTAACAGAA |
| Dll4 F | GACCTGCGGCCAGAGACTT |
| Dll4 R | GAGCCTTGGATGATGATTTGG |
| Rbpsuh F | GCGGATAAAGGTCATCTCCA |
| Rbpsuh R | TTCCTGAAGCAATGCACAAG |
| Hes1 F | CCCCAGCCAGTGTCAACAC |
| Hes1 R | TGTGCTCAGAGGCCGTCTT |
| Hes3 F | AAGGGAGCAGAAAAGCATCA |
| Hes3 R | CTATGGCAGGGAGCTTTGAG |
| Hes5 F | TGGGCACATTTGCCTTTTGT |
| Hes5 R | CAGGCTGAGTGCTTTCCTATGA |
| Hes6 F | GAAGTGGCCAATCTTGAGACTGA |
| Hes6 R | GGATTGCTGTGGCCTGTGT |
| HeyL F | AGATGCAAGCCCGGAAGAA |
| HeyL R | CGCAATTCAGAAAGGCTACTGTT |
| Hey1 F | GGGAGGGTCAGCAAAGCA |
| Hey1 R | GCTGCGCATCTGATTTGTCA |
| Hey2 F | CACATCAGAGTCAACCCCATGT |
| Hey2 R | GTGAGGAGAGCAGAGCCATGA |
| Lfg F | CGAGCACAAAGTGAGACCTG |
| Lfg R | TGCCGTGCTCATGAAGTGTC |
| Mfg F | AGACTACCTGGGCCTTCCAT |
| Mfg R | TGAATGTCTGTTGCCTGATCCT |
| Mib2 F | CTAGGATGGCAGAGATGGGC |
| Mib2 R | ACCCAGAAGCTGTTGTGCTT |
| Neurb1 F | AATCGTCTCTGGTGACAGCC |
| Neurb1 R | GTGCACTCTCCATTCCTGCT |
| Ascl1/Mash1 F | CCAACTACTCCAACGACTTGAAC |
| Ascl1/Mash1 R | TCCTGCCATCCTGCTTCCAAAG |
| Bcl2 F | TGGGATGCCTTTGTGGAACT |
| Bcl2 R | CAGCCAGGAGAAATCAAACAGA |
| Bax F | CCAAGAAGCTGAGCGAGTGTCT |
| Bax R | AGCTCCATATTGCTGTCCAGTTC |
| Apaf1 F | GGGTGGGTCACCATCTATGG |
| Apaf1 R | TTCCGCAGCTAACACAGACTTG |
| Trp53 F | AGCGCTGCTCCGATGGT |
| Trp53 R | TTCCTTCCACCCGGATAAGA |
| p53 F | CTCTCCCCCGCAAAAGAAA |
| p53 R | CGCCCGCGGATCTTG |
| Cdkn1c F | CAATCAGCCAGCAGAACAGC |
| Cdkn1c R | CAGCTCCTCGTGGTCTACAG |
| Gapdh F | AGGTCGGTGTGAACGGATT |
| Gapdh R | TCTAGACCATGTAGTTGAGGTCA |
